# Computational Analysis Reveals Monomethylated Triazolopyrimidine as a Novel Inhibitor of SARS-CoV-2 RNA-Dependent RNA Polymerase (RdRp)

**DOI:** 10.3390/molecules27030801

**Published:** 2022-01-26

**Authors:** Anandakrishnan Karthic, Veerbhan Kesarwani, Rahul Kunwar Singh, Pavan Kumar Yadav, Navaneet Chaturvedi, Pallavi Chauhan, Brijesh Singh Yadav, Sandeep Kumar Kushwaha

**Affiliations:** 1 Bioinformatics, DBT-National Institute of Animal Biotechnology (NIAB), Hyderabad 500032, India; karthic28199@gmail.com (A.K.); veerbhan8586@gmail.com (V.K.); 2 Amity Institute of Biotechnology, Amity University Mumbai, Navi Mumbai 410206, India; 3 Hap Biosolutions, Pvt. Ltd., Bhopal 462042, India; 4 Cyano Biotech Lab, Department of Microbiology, School of Life Sciences, Hemvati Nandan Bahuguna Garhwal University, Srinagar (Garhwal) 246174, India; rksingh.hnb@gmail.com; 5 Department of Veterinary Physiology and Biochemistry, Faculty of Veterinary and Animal Sciences, Banaras Hindu University, Mirzapur 231001, India; pavan.yadav@bhu.ac.in; 6 Department of Molecular and Cell Biology, Henry Wellcome Building, University of Leicester, Leicester LE1 7RH, UK; 14.navneet@gmail.com; 7 Department of Biology, Lund University, 22370 Lund, Sweden; pallavi.chauhan@biol.lu.se; 8 Faculty of Biosciences and Aquaculture, Nord University, N-8049 Bodø, Norway

**Keywords:** SARS-CoV-2, triazolopyrimidine, RNA-dependent RNA polymerase (RdRp), essramycin, non-structural proteins (NSP), Remdesivir, Favipiravir

## Abstract

The human population is still facing appalling conditions due to several outbreaks of Severe Acute Respiratory Syndrome Coronavirus-2 (SARS-CoV-2) virus. The absence of specific drugs, appropriate vaccines for mutants, and knowledge of potential therapeutic agents makes this situation more difficult. Several 1, 2, 4-triazolo [1, 5-a] pyrimidine (TP)-derivative compounds were comprehensively studied for antiviral activities against RNA polymerase of HIV, HCV, and influenza viruses, and showed immense pharmacological interest. Therefore, TP-derivative compounds can be repurposed against the RNA-dependent RNA polymerase (RdRp) protein of SARS-CoV-2. In this study, a meta-analysis was performed to ensure the genomic variability and stability of the SARS-CoV-2 RdRp protein. The molecular docking of natural and synthetic TP compounds to RdRp and molecular dynamic (MD) simulations were performed to analyse the dynamic behaviour of TP compounds at the active site of the RdRp protein. TP compounds were also docked against other non-structural proteins (NSP1, NSP2, NSP3, NSP5, NSP8, NSP13, and NSP15) of SARS-CoV-2. Furthermore, the inhibition potential of TP compounds was compared with Remdesivir and Favipiravir drugs as a positive control. Additionally, TP compounds were analysed for inhibitory activity against SARS-CoV RdRp protein. This study demonstrates that TP analogues (monomethylated triazolopyrimidine and essramycin) represent potential lead molecules for designing an effective inhibitor to control viral replication. Furthermore, in vitro and in vivo studies will strengthen the use of these inhibitors as suitable drug candidates against SARS-CoV-2.

## 1. Introduction

The SARS-CoV-2 virus is still developing new variants that cause infection, such as Omicron, the most recent global SARS-CoV-2 variant [1]. Furthermore, the lack of rapid, responsive, and inexpensive diagnostic methods for SARS-CoV-2 infection are posing more severe challenges to humanity [2]. Despite immense drug development, there are currently no commercially licensed drugs available, except for Remdesivir, 2-DG, and pegylated Interferon alpha-2b, to combat against the SARS-CoV-2 virus [3,4,5]. Every day, the virus infects millions of people and causes approximately 10,000 deaths worldwide. In April 2021, rates of infection and fatalities climbed steadily, especially in India. This is highly concerning because the human population remains vulnerable in many countries, even after massive vaccination drives. Therefore, the concomitant usage of multiple therapeutic options can help to reduce the disease burden [6,7].

SARS-CoV-2 is a single-stranded, positive-sense RNA virus of the betacoronaviridae family and a beta coronavirus family member [8,9]. The 30 kb RNA genome of human coronavirus encodes 20 proteins (4 structural and 16 non-structural). Most of them are part of transcription, replication or proteolytic activities [10]. The spike protein of SARS-CoV-2, one of the essential structural proteins, is involved in host cell recognition, attachment, and entry [11], whereas most of the non-structural proteins (nsp1–16) are involved in the virus’s replication cycle. RNA-dependent RNA polymerase (RdRp), a protein 932 amino acids in length, is a vital component of virus replication. It has been used as a drug target to inhibit the replication of various viruses, such as Zika and HCV virus, due to its conserved binding domain residues [12,13]. Targeting the critical catalytic amino acid residues of the RdRp protein (Ser759-Asp760-Asp761 and Asp618) can inhibit the replication activity of SARS-CoV-2 [14]. The conserved RdRp active site residue positions of SARS-CoV-2 is analogous to other RNA polymerases [15,16]. Thus, it is the most promising therapeutic target for antiviral drugs [17]. In this scenario, the in silico screening of already known compounds against RdRp may identify potential repurposable drug candidates against the highly contagious infection of COVID-19 [18].

In this context, 1, 2, 4-triazolo [1, 5-a] pyrimidine (TP) compounds, and their derivatives can help us to identify lead compounds against COVID-19. Several TP-derivative compounds were synthesised and screened for therapeutic applications like antibacterial, antifungal, antiparasitic, anticancer and antiviral [19,20] acting as nucleoside analogues. Recently, synthetic triazolopyrimidine derivatives were identified as viral polymerase inhibitors against HCV, HIV, and influenza viruses [20,21]. However, only two natural TP derivatives were reported in the literature. The first natural TP derivative, essramycin, was isolated from a marine organism, *Streptomyces sp. Merv 8102* and the second, 7-methyl-6-nitro [1,2,4] triazolo [1, 5-a] pyrimidin-5-ol (MTP), was identified in the non-polar extract of a thermophilic cyanobacterium, *Leptolyngbya* sp. HNBGU 002 [22]. Both natural TP-derivative compounds were reported to exhibit antibacterial and antiviral activities [20,21]. These studies of TP-derivative compounds and their understanding encouraged us to explore their inhibitory activity against RdRp enzymes and non-structural protein targets of the SARS-CoV-2.

In this study, we computationally evaluated the inhibition potential of four TP-based ligands, one parent TP compound (triazolopyrimidine (TPP-1), two natural derivatives (essramycin (EMC-1) and monomethylated triazolopyrimidine (Comp-1)), and one synthetic TP derivative (MTP stereoisomer (TBP-2)) against RdRp and non-structural proteins of SARS-CoV-2. Subsequently, molecular dynamics (MD) simulation was performed on the best-scoring TP-ligands complexes with SARS-CoV-2 and SARS-CoV RdRp proteins to comparatively assess their stability, interactions and specificity. Further, Remdesivir (REM) and Favipiravir (FP) were used as positive controls against the RdRp protein of SARS-CoV-2 and SARS-CoV to compare the inhibition efficacy of TP-derivative compounds.

## 2. Materials and Methods

### 2.1. Meta-Analysis to Explore Genomic Variability of RdRp Protein as a Potential Drug Target

Genomic variation analysis is an essential criterion for selecting drug targets to find potential inhibitor molecules. To explore the genomic variation in the RdRp protein of SARS-CoV-2 from experimental data, ProteomeXchange, a proteomics repository was explored, and the proteome data of two human cell-line-based proteomes (PXD017710, PXD018581) and a COVID-19 patient sample (PXD021328) were selected for processing using Proteome Discoverer 2.4 (Thermo Scientific, Waltham, MA, USA). For this purpose, ORF1ab protein sequences were extracted from the NCBI database. A local stand-alone BLAST [23] was set up to find similar sequences at 95% sequence similarity cut-off using Wuhan-Hu-1 isolates of SARS-CoV-2 (MN908947.3) RdRp protein sequences as a reference. A Perl script was used to extract the RdRp protein region (4393–5324) from the downloaded orf1ab protein FASTA file. All the sequences with more than 0.1% ambiguous (“X”) characters were removed from the study to decrease the possibility of incomplete or low-quality sequences. Identical sequences were merged into single clusters using CD-HIT [24] with 100% sequence identity, and clustered non-redundant sequences were used as a reference in proteomics analysis to identify RdRp peptides from experimental data. The above-mentioned proteomics raw files and processed reference sequences were used for peptide identification at a 5% false discovery rate (FDR). Parameters of maximum missed cleavages (1), allowed mismatch (1), charge range (2–3), and *m/z* range (396–1600) were set for peptide search. The mass tolerance of 10 ppm and 0.8 Dalton (Da) was selected for parent ions and fragment ions matching, respectively, and the remaining search parameters were set at default. Additionally, multiple sequence alignment (MSA) was performed upon downloaded RdRp sequences using ClustalW [25] (version 1.2.4), and a consensus sequence was generated to discover high confidence amino-acid substitution between reference and downloaded sequences. Available RdRp protein structures, apo-RdRp (PDB ID: 7BV1) and Remdesivir-bound RdRp (PDB ID: 7BV2) were evaluated to verify the effect of mutation on structural conformation, molecular stability and flexibility using DynaMut server [26]. Furthermore, a phylogenetic analysis was also performed to explore the conserved nature of the RdRp protein among several respiratory disease-causing viruses, such as respiratory syncytial virus, rhinovirus, MERS and SARS-CoV. Amino acid sequences of viral RdRps were retrieved from the UniProt database and aligned using the Mafft tool of the NGPhylogeny.fr web server. Phylogenetic trees were generated with 1000 bootstrap replicates via FastME Balanced Minimum Evolution-Subtree Pruning and Regrafting (BalME-SPR) [27]. iTOL was used to visualise and draw the trees [28].

### 2.2. RdRp Protein and Ligand Structures Preparation

The structures of four TP ligands—synthetic triazolopyrimidine (TPP-1), essramycin (EMC1), monomethylated triazolopyrimidine (Comp-1) and MTP stereoisomer (TBP-2)—capable of eliciting antiviral activity were prepared for computational molecular docking using ChemSketch tool [29]. Generated 2D SDF files of all four compounds were converted into 3D PDB file format using the Open Babel tool [30]. These ligands were used for docking without any further modifications. The same procedure was followed for Favipiravir and Remdesivir. Three-dimensional structures of SARS-CoV-2 and SARS-CoV RdRp proteins were obtained from the RCSB protein structure database [31]. The electron microscopy-based protein structure of SARS-CoV-2 RNA-dependent RNA polymerase, in complex with Remdesivir (PDB ID: 7BV2), was selected for this study [3], whereas PDB ID 6NUR was selected for the RdRp protein structure of SARS-CoV-2 virus. The protein preparation wizard of Discovery Studio 2020 was used to prepare the structure of RdRp by removing water molecules and adding polar hydrogen atoms. Other small molecules such as ligands and metal ions previously bound to the protein were also removed. Similarly, protein structures of non-structural proteins of SARS-CoV-2 (NSP1 (7K7P), NSP2 (7MSX), NSP3 (7CZ4), NSP5 (5RHB), NSP8 (6WTC), NSP13 (5RLJ) and NSP15 (7KEH)) were downloaded from RCSB database and prepared for the docking. The extracted protein structures were further used in docking and molecular dynamics simulation studies.

### 2.3. Molecular Docking and Simulation of RdRp with Triazolopyrimidine Derivative Compounds

Molecular docking of the four TP ligands was performed against RdRp and NSPs to identify a putative binding site, binding interactions and affinity. The protein structures of RdRp of SARS-CoV-2 and SARS-CoV, non-structural proteins and a combination of four TP ligands were submitted to DockThor tool for blind docking on default settings [32]. To better understand the behaviour of the apo RdRp (SARS-CoV-2 and SARS-CoV) and docked complexes (SARS-CoV-2 RdRp/Comp-1/EMC-1 and SARS-CoV RdRp/Comp-1/EMC-1) under dynamic biological conditions, atomistic MD simulations were performed using CHARMM36 force field in GROMACS 2020.3 package [33]. CGenFF server was employed to generate topology files of ligands (EMC-1 and Comp-1) [34]. The docked complex was placed in the centre of a dodecahedron box, with the periodic boundary condition (PBC) set to 2 nm from each face. The box around the protein–ligand complexes was filled using a simple point charge (SPCE) water model, and the system was neutralised by replacing equivalent numbers of water molecules with exact counter ions (Na^+^). The steepest descent algorithm was used to minimise the system’s energy until tolerance of 1000 kJ/mol/nm was reached. Then, the equilibrated systems were subjected to 50 nanoseconds (ns) production run. Additional runs were performed for 100 ns on SARS-CoV-2 RdRp/ligand complexes to strengthen our findings. The random velocity of the first and second runs was set to −1 and −2, respectively. All the results were generated and analysed using Xmgrace. Further g_mmpbsa script [35] was used to compute the MM-PBSA binding free energies. Furthermore, docking and MD studies were also performed for Remdesivir and Favipiravir drugs against RdRp protein of SARS-CoV-2 and SARS-CoV to explore the inhibition potential of TP-derivative compounds.

## 3. Results

The world is still experiencing the COVID-19 pandemic due to the evolution of human coronavirus spreading infection waves worldwide, highlighting the serious need to develop therapeutic drugs [3]. The success of drug discovery is critically dependent on selecting the appropriate therapeutic drug targets and targeting compounds. Therefore, a deep understanding and knowledge of selected targets and compounds are required to accelerate drug-discovery processes. Various research studies have been performed since the pandemic began in late 2019. However, none of these studies explored the potential of triazolopyrimidine or its derivatives against SARS-CoV-2 [36,37,38]. In this study we perform the genomic variability assessment of the RdRp protein, molecular docking, multiple simulation-based evaluation of four triazolopyrimidine-derivative compounds and two used drugs (Remdesivir and Favipiravir) against the SARS-CoV-2 RdRp protein [39].

### 3.1. Meta-Analysis of the Genomic Variation in RdRp Protein

The knowledge of genomic variations is an important criterion for drug target selection to inhibit viral infection. However, most of the performed studies were focused on vaccine development, disease diagnosis and host–pathogen interaction, rather than the exploration of genomic variations in the RdRp protein. Therefore, we performed a literature survey to explore the genomic variation of the RdRp protein (Supplementary Materials Table S1). In general, the RdRp protein is a highly conserved viral protein. However, the recent outbreak of the SARS-CoV-2 virus motivated us to explore its most updated genomic variation from publicly available resources such as NCBI. For this purpose, 39465 orf1ab sequences of SARS-CoV-2 were downloaded, and after processing, 6377 RdRp sequences were used as a reference in proteomics analysis. As a result, a high-level expression of RdRp was observed in SARS-CoV-2-infected cell lines and patient studies (Supplementary Table S2). All of the identified RdRp peptides were precisely matched to publicly available proteins. The presence of multiple RdRp peptides in the same sample and the same peptides in multiple samples without any variation, corroborate the presence of the RdRp protein in biological samples (Table 1), reflecting the appropriateness of the RdRp protein as a potential drug target for treating COVID-19 infection. Reference RdRp sequences (6377) were also analysed through MSA and consensus sequence generation. In the reference RdRp protein sequence analysis, most amino acids were found highly conserved, but only a few amino acid substitutions were observed across the RdRp sequence (Supplementary File S2 RdRP-MSA). However, the number of sequences with the amino acid substitution at a specific position was insignificant compared to the total number of sequences. Occasionally, sequencing or technical processing may also cause sequence variations. Therefore, a consensus sequence was generated at 50% sequence cut-off using multiple sequence alignment of reference RdRp sequences to explore the high-confidence sequence variation. As a result, a high-confidence sequence variation was found at the 323rd amino acid position, where proline was replaced by leucine. It was also observed that this mutation was more frequent in sequences deposited from Europe, America and India [40].

**Figure 1:**
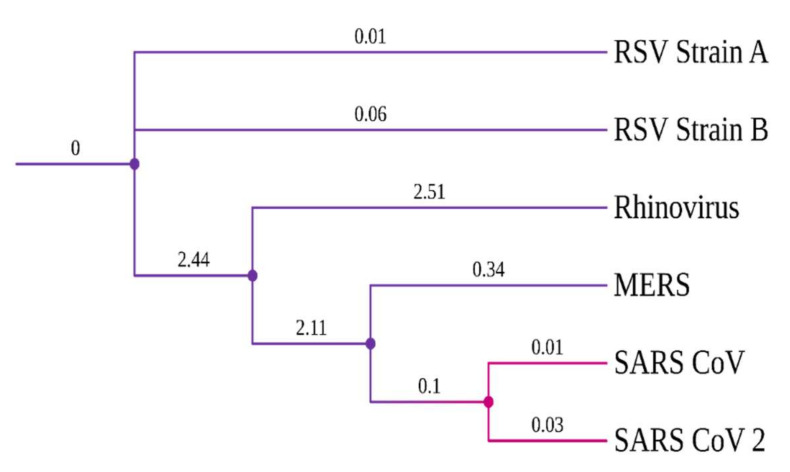
Phylogenetic tree of viral RdRp proteins.

Evolutionary conserveness is another crucial criterion for drug target selection. Therefore, a phylogenetic analysis was conducted to investigate the evolutionary characteristics of the SARS-CoV-2 RdRp protein among six other viruses that cause COVID-19-like respiratory symptoms/diseases (Table S3). SARS-CoV viruses have a common origin and are closely related to the highly contagious viruses, MERS and Rhinovirus (Figure 1). Notably, SARS-CoV-2 and SARS-CoV RdRp proteins have a high degree of sequence conservation. However, amino acid variations were observed due to differences in RdRp protein length. Nonetheless, the success of the TP-derivative compounds, against the RdRp protein of the influenza virus [21,41], evoked an interest in testing them against the RdRp protein of SARS-CoV-2.

**Figure 2:**
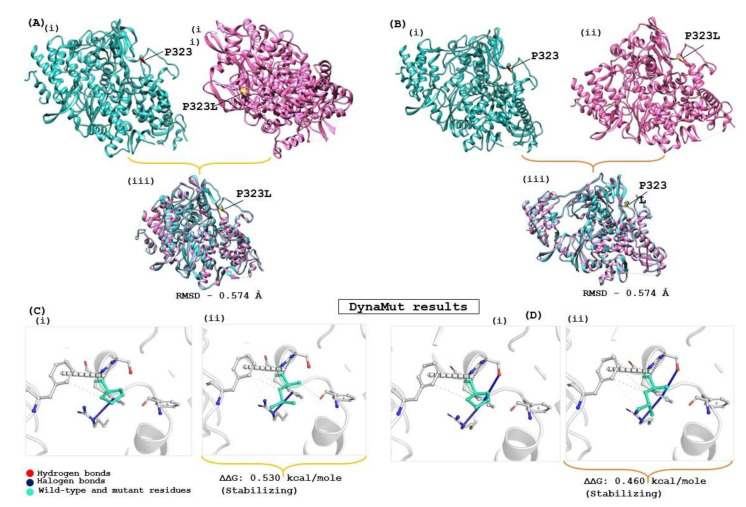
Evaluation of the impact of the mutation on RdRp protein of SARS-CoV-2. (**A**) Apo RdRp protein (7BV1), and (**B**) Apo form of Remdesivir complex RdRp (7BV2); (**i**) represent the wild form, (**ii**) mutated form and (**iii**) superimposed structure of both proteins. (**C**,**D**) show the interatomic interaction by P323L of RdRp protein; (**Ci**,**Di**) represent the wild type of RdRp protein, (**Cii**,**Dii**) show proline to leucine substitution at 323 position.

The structural stability of the drug target is highly influenced by genomic variation in protein sequences. Therefore, the effect of the identified mutation (P323L) was evaluated on the available RdRp protein structures (7BV1 and 7BV2) using the DynaMut server, which provides information about how a mutation in the native protein structure can affect protein stability and flexibility. According to the DynaMut result, the wild type and mutant RdRp proteins of SARS-CoV-2 have different vibrational entropy energies (ΔΔSVibENCoM) (7BV1: −0.252 & 7BV2: −0.320 kcal·mol^−1^·K^−1^); a negative vibrational entropy energy represents a reduced protein flexibility. In contrast, the positive vibrational entropy energy represents an increase in flexibility, and the ΔΔG value below zero indicates protein destabilisation, whereas a ΔΔG value above zero indicates an increase in protein stability due to mutation [26]. Dynamut results reveal that the P323L mutation reduces the mutant protein’s molecular flexibility and enhances protein stability (ΔΔG for the P323L mutant; 7BV1:0.530 kcal/mol and 7BV2:0.460 kcal/mol). When both apo forms of RdRp of SARS-CoV-2 (7BV1 and 7BV2) were compared, a structural deviation of 0.574 Å was estimated. A RMSD value 0.574 Å was found when wild and mutant protein structures (7BV1 and 7BV2) were compared individually for both proteins (Figure 2). Despite the increasing protein stability, P323L mutation did not cause any structural deviation in the native protein structure. However, when REM was bound, the mutated RdRp acquired a more compact and tighter packing, which might be the reason for its increased binding affinity, revealed in a computational simulation study [42]. Our study also had similar observations (Supplementary Table S5). Hence, for the subsequent analysis, FP and REM drugs were used to compare the inhibition potential of TP derivatives due to known experimental information of molecular binding and interaction.

### 3.2. Interaction Analysis and Selection of Lead Compounds as SARS-CoV-2 RdRp Inhibitors

TP derivatives exhibit antibacterial and antiviral properties against several pathogenic agents. This study analysed four TP-derivative compounds for their binding affinity and intermolecular interactions against the SARS-CoV and SARS-CoV-2 RdRp proteins. The binding affinities of the four ligands ranged from approximately −6.13 to −7.25 kcal/mol, whereas their total energies ranged from −3.6 to −13.86 kcal/mol. Comp-1 and EMC-1 were found in the top two ligands in docking studies due to their high binding affinity and low total energy. A comparative docking study was also performed with two tested drugs (Remdesivir and Favipiravir) against the SARS-CoV-2 and SARS-CoV RdRp proteins [43,44]. The total energy of Remdesivir renders the complex of SARS-CoV-2–RdRp more stable, and hence the usage of this drug against SARS-CoV-2 infection has been favoured, over the last one and half years. However, the binding affinity of the drugs used in this study is comparable to REM, whereas, in terms of both binding affinity and total energy, Comp-1 and EMC-1 outperform FP, TP ligand structures mimic the nucleosides, and these structures are effectively accommodated at the surface SARS-CoV-2 and SARS-CoV RdRp. A comparison of binding affinity and total energy is provided in Table 2.

**Figure 3:**
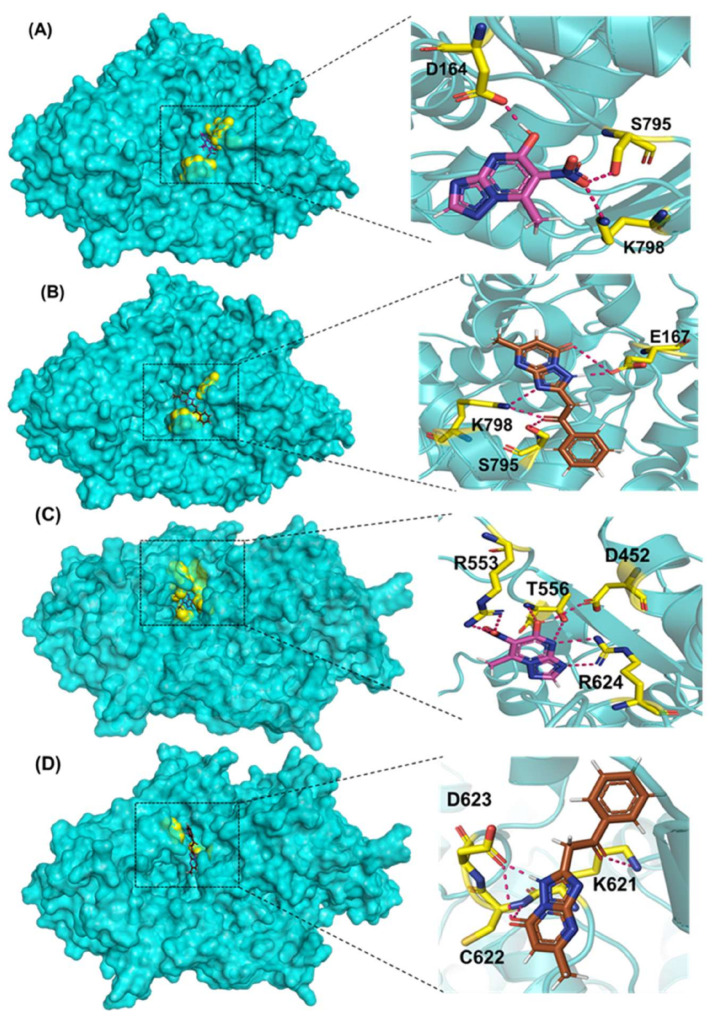
Three-dimensional visualisation of docked complexes and protein–ligand hydrogen bond interactions: (**A**) SARS-CoV-2 RdRp/Comp-1, (**B**) SARS-CoV-2 RdRp/EMC-1, (**C**) SARS-CoV RdRp/Comp-1, and (**D**) SARS-CoV RdRp/EMC-1. Please refer to Supplementary Figure S2 for FP and REM–ligand interactions with SARS-CoV-2 RdRp and Figures S3 and S4 to visualise protein–ligand interactions of the individual complex.

Comp-1 and EMC-1 showed hydrogen bonding (H bond) with the RdRp (SARS-CoV-2) residues, Lys798 and Ser795. Comp-1 exhibited pi-anion, pi-alkyl, and hydrogen bonding with Glu167, Val166 and Asp164, respectively. EMC-1 only had one added H bond with Glu167 (Figure 3A,B). These interactions yielded the binding energy of −6.134 and total energy of −8.308 kcal/mol in the Comp-1–RdRp complex. Similarly, −6.283 and −5.416 kcal/mol were the binding affinities and total energy of the EMC-1–RdRp complex. The TP-derived compounds also demonstrated high interactions with the SARS-CoV RdRp protein, possibly due to the structural similarity, sequence conservation and sharing of similar binding sites between SARS-CoV-2 and SARS-CoV RdRps. To understand the binding analogy of TP-derived compounds, the protein structure of RdRp of SARS-CoV-2 and SARS-CoV were compared, and a structural deviation of 0.657 Å was estimated (Figure S1A). Moreover, the docking studies showed that the binding sites of the TP-based ligands almost overlapped with FP. Ser795 and Lys798 amino acids were common interactants between EMC-1 and FP, indicating that the Comp-1 and EMC-1 would have a similar action mechanism to Favipiravir. On the other hand, REM had a different binding site and energy.

**Figure 4:**
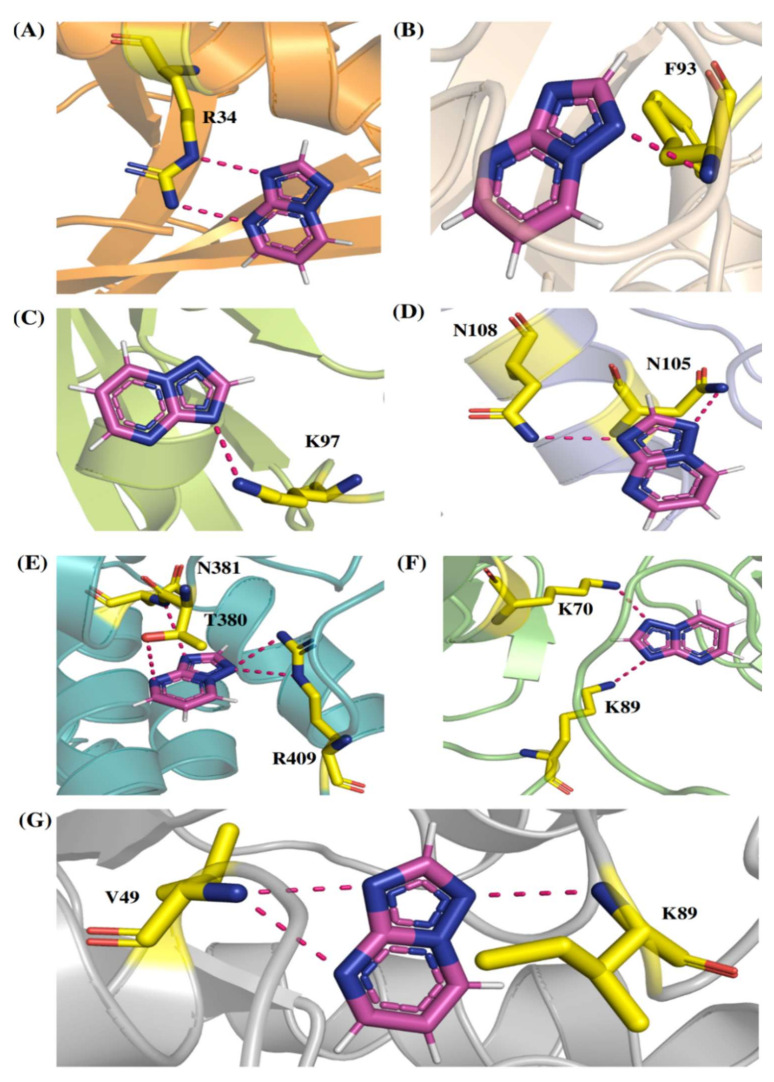
The 3D interactions of Comp-1-NSP docked complexes. (**A**) NSP1, (**B**) NSP2, (**C**) NSP5, (**D**) NSP8, (**E**) NSP13, (**F**) NSP15 and (**G**) NSP3.

Comp-1 displayed a total energy of −13.864 kcal/mol with a binding affinity of −6.314 kcal/mol, reflecting the stability of interactions. With the total energy of −6.524 kcal/mol and binding affinity of −7.039 kcal/mol, EMC-1 also interacted well with RdRp. Both the ligands bound at the same interaction pocket of RdRp. EMC-1 shows three hydrogen bonds with Asp623, Lys621 and Asp623: a pi–cation interaction with Arg553. On the other hand, Comp-1 had five hydrogen bonds with the residues Arg553, Asp542, Thr556 and Arg624. Additionally, the Asp623 residue interacted with the ring structures of Comp-1 via pi–anion interactions (Figure 3C,D). The 3D representation of SARS-CoV-2 RdRp interactions with Favipiravir and Remdesivir are provided in the Supplementary File, Figure S2. In Figure 3, only hydrogen bonds are shown. Other interactions among docked complexes are presented in Supplementary Files (Figures S3 and S4). Additionally, the four TP ligands also docked with the NSP1, NSP2, NSP3, NSP5, NSP8, NSP13 and NSP15 protein structures of SARS-CoV-2 (Figure 4). Comp-1 manifested the best binding and total energies in all seven complexes (Table 3). A detailed description of the docking of other non-structural proteins with other TP-derivative compounds is given in Supplementary File S1 (Table S4). Moreover, the TP-based compounds such as monomethylated triazolopyrimidine and essramycin showed high interactions with many other non-structural proteins involved in the life cycle, replication and transcription of the COVID-19 virus. Aside from the hydrogen bonds formed by the ligand Comp-1 with the SARS-CoV-2 NSPs, multiple other pi–alkyl, pi–pi and pi–sigma interactions are provided in Supplementary Figure S5.

The ligands TBP-2 and TPP-1 also showed a good binding affinity with the SARS-CoV-2 RdRp. However, both compounds possessed a lower total energy and had fewer interactions than the top two ligands (Comp-1 and EMC-1). Overall, RdRp of SARS-CoV-2 and SARS-CoV had better interaction and stability with the ligands, Comp-1 and EMC-1. Therefore, both the compounds were thoroughly studied through a molecular dynamics simulation analysis.

### 3.3. MD Simulation of Apo RdRp and Docked Ligand Complexes

**Figure 5:**
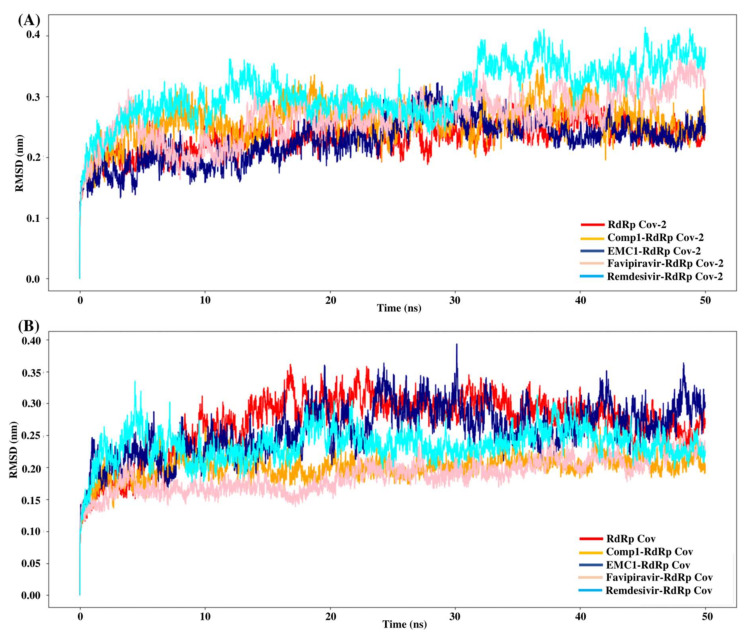
RMSD values of SARS-CoV-2 (**A**) and SARS-CoV (**B**) apo RdRp and docked complexes during 50 ns MD simulation.

Molecular dynamics simulation is a computational approach to studying biomolecules’ structural stability and molecular behaviour. The MD simulation of the unbound RdRp protein and docked complexes of RdRp–ligands was performed at 100 ns using GROMACS software. High-binding-affinity complexes of TP ligands and RdRp proteins (SARS-CoV-2 RdRp/Comp-1/EMC-1 and SARS-CoV RdRp/Comp-1/EMC-1) were selected for analysis, such as root mean square deviations (RMSD), root mean square fluctuations (RMSF), solvent-accessible surface area (SASA) and radius of gyration (R_g_). The interaction between the RdRp protein and TP ligands was determined based on binding energy and the number of hydrogen bonds formed. The MD simulation results show that the unbound RdRp displayed more RMSD fluctuations than the complexes. The RMSD of the ligand-bound SARS-CoV-2 RdRp complexes fluctuated until 30 ns; after this, it plateaued (Figure 5A), reflecting the conformational changes in RdRp in the presence of the inhibitor ligand before the final structural rearrangements to attain the best pose. The fluctuations of ligands compared, i.e., REM and FP, were higher than those of Comp-1 and EMC-1. This suggested that the TP ligands stabilised before REM and FP. The average RMSD of the Comp-1 complex was approximately 0.24 nm and 0.22 nm for the EMC-1 complex. The RMSD profile of Comp-1 was quite similar and stable with both RdRp structures. EMC-1 was observed to be more stable when complexed with SARS-CoV-2 RdRp than SARS-CoV RdRp. In general, the RMSD values of the ligand complex remained more stable than the apo form throughout the simulation period, especially with Comp-1-RdRp-CoV (Figure 5B). This indicated that these ligands had a high potential as inhibitors due to their stable complex formation characteristics. The RMSD of Comp-1 and EMC-1 was wavering until 30 ns, and then it attained a steady deviation and remained stable up to 100 ns simulations. Despite using different random velocities, similar RMSD patterns were found (Figure S6). Moreover, Comp-1 had a similar RMSD profile as REM and FP. However, the RMSD of REM increased to an average of 0.42 and was different from the previous two runs.

**Figure 6:**
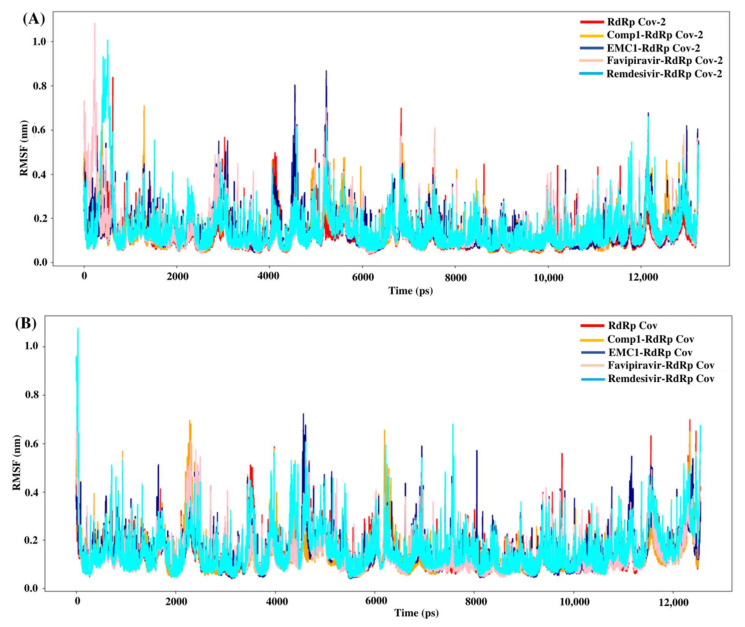
RMSF values of SARS-CoV-2 (**A**) and SARS-CoV (**B**) apo RdRp and docked complexes during the 50 ns MD simulation.

The root mean square fluctuation (RMSF) values represent each residue’s thermodynamic stability and degree of movement. Smaller RMSF values indicate a more stable region, while larger RMSF values indicate a more flexible region. In general, the apo RdRp did not illustrate large fluctuations, possibly due to its fixed state in the absence of a ligand. However, upon ligand recognition, the flexible residues in the ligand-binding regions fluctuated to accommodate ligands to stay in equilibrium. Most of the time, RMSF values of Comp-1 remained between 0.1 to 0.5 nm, while the EMC-1 ranged from 0.1 to 0.6 nm (Figure 6A). The RMSF of EMC-1 complex at specific time points (around 5000 ps and 12,000 ps) fluctuated the most, where Comp-1 complex stayed relatively unchanged.

In comparison, FP and REM initially had higher RMSF values, and then stabilised, whereas other ligand complexes, including the unbound RdRp had fewer fluctuations. The 100 ns run at the random velocity −1 also had similar RMSF profiles, except at the initial stages where the RdRp’s residues increased the fluctuations to accommodate for the drug. At the values of ~4900 ps and 7800 ps, the EMC-1 complex showed higher fluctuations to keep the complex intact. In the run with a random velocity of −2, only the REM–RdRp complex had higher RMSF values than in the initial period. Otherwise, all of them had very few fluctuations. In the case of SARS-CoV RdRp, Comp-1 and EMC-1 had similar RMSF profiles indicating that both were very efficiently accommodated at the binding site (Figure 6B). Additionally, the RMSF profile of FP and REM are similar to the ligands, Comp-1 and EMC-1, the only outlier being the initial stage, where the RMSF value of REM reached above 1 nm.

**Figure 7:**
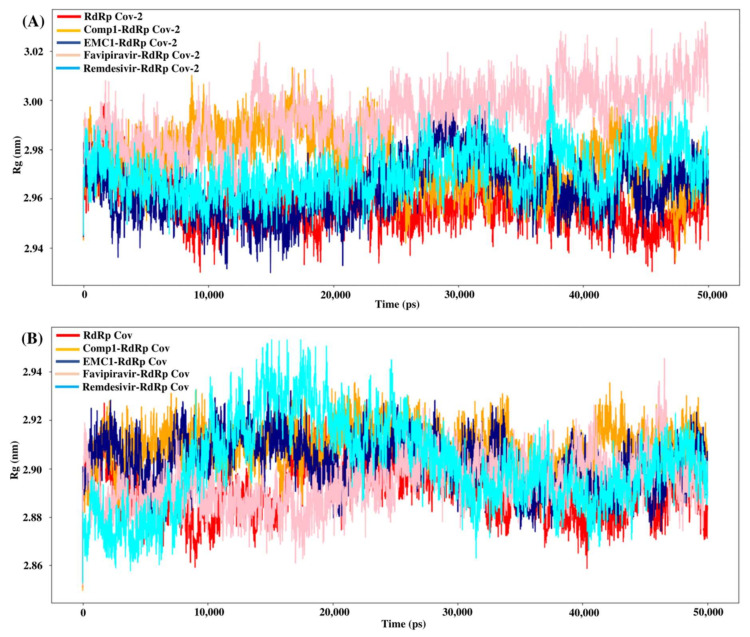
R_g_ values of SARS-CoV-2 (**A**) and SARS-CoV (**B**) apo RdRp and docked complexes during 50 ns MD simulation.

The radius of gyration or gyradius (R_g_) analysis reveals the stability of each molecule and the overall dimension and compactness of the structure. The R_g_ of SARS-CoV-2 RdRp complexed with Comp-1 was relatively high up to 30 ns. However, after that, it stabilised. In contrast, apo the RdRp and EMC-1 complex was stable almost throughout the simulation. Overall, the R_g_ of the protein–ligand complex varied from 2.94 to 3.02 nm (Figure 7A). This range of R_g_ was very close to the average R_g_ value (2.96 nm) of apo RdRp. The average gyradius of REM was comparable to Comp-1 and EMC-1, but FP had a slightly higher Rg value (3 nm). The TP-derivative-based complexes had a stable and similar Rg profile in the 100 ns runs. FP had an average Rg of 2.96 in both runs; on the other hand, the Rg of REM was an outlier in the second run. In the case of SARS-CoV RdRp, the complexed proteins fluctuated in the Rg range of 2.88 to 2.92 nm (Figure 7B), and the unbound form of RdRp had an R_g_ range of 2.86 to 2.92 nm. Even though FP had a similar gyradius range as the ligands studied in this work, the REM Rg range was scattered from 2.86 nm to 2.96 nm. The binding of the ligand could cause a rise in the gyradius values. Later, the protein complex attained a stable conformation similar to the unbound form of the protein.

In conclusion, during the 50 ns MD simulation, the Comp-1 and EMC-1 RdRp complexes became stable after an initial period of molecular adjustment with the ligands. Furthermore, 100 ns simulations were performed to analyse the behaviour of the ligands with the SARS-CoV-2 RdRp as an inhibitor. The solvent-accessible surface area (SASA) of a protein indicates an area of the protein that is exposed to the solvent. Hence, the binding of a ligand can influence the solvent-accessible area. In the first run, EMC-1 had a slightly lesser average of SASA (~370 nm^2^), whereas Comp-1 had an average SASA of 370 nm^2^ in the second run. In both runs, unbound RdRp had a lower SASA, FP had almost equal values, and REM had a higher SASA than Comp-1 and EMC-1.

The number of intermolecular hydrogen bonds (H bonds) formed by the protein and ligand is another factor that can aid in the stability of the complex. REM followed by FP formed the highest number of H bonds, whereas Comp-1 and EMC-1 constantly maintained 2–3 H bonds. The difference in hydrogen bonds might be due to the ligands’ chemistry, size, and configuration.

The binding free energy of the top complexes was deduced using the MM-PBSA method (Formula 1); ΔG_binding_ denotes the overall binding energy of the complex, whereas G_receptor_ denotes the binding energy of a free receptor, and G_ligand_ denotes the binding energy of an unbounded ligand:

ΔG_binding_ = G_complex_ − (G_receptor_ + G_ligand_) (1)

The average binding energy of the Comp-1/SARS-CoV-2 RdRp complex is −31.53 kcal/mol and −33.165 with the EMC-1 complex after two runs of 100 ns MD simulation. The FP complex stands at −17.7 kcal/mol. However, REM has an average binding energy of −39.74 kcal/mol.

## 4. Discussion

The current pandemic is of serious concern because new SARS-CoV-2 variants and reinfection cases are continuously reported worldwide, even after massive vaccination drives. In this regard, a multi-pronged approach was adopted to handle SARS-CoV-2 virus infection, which included social distancing, working from home, regular testing, vaccinating and immunity building. The concurrent usage of drugs such as Remdesivir and 2-deoxy-D-glucose are helping to fight against COVID-19 [4]. However, the discovery of new drugs is necessary to increase the effectiveness and affordability of treating the disease. In the literature, synthetic derivatives of 1,2,4-triazolo(1–5-a) pyrimidine (TP), nucleoside analogues were reported as RNA polymerase inhibitors against various viruses, such as hepatitis C virus (HCV), human immunodeficiency virus type-1 (HIV) and influenza virus [20]. This study repurposed the TP-derivative compounds to inhibit viral replication in the host. SARS-CoV-2 RdRp has a high sequence similarity with RdRp of influenza virus (Figure 1). Hence, it is highly probable that these TP derivatives could be helpful for inhibiting the spread of the coronavirus that causes COVID-19.

Recent evolution and genomic variations in SARS-CoV-2 viral strains were reported in various research studies [45,46]. However, most studies focused on exploring genomic variations in spikes, enveloping, membranes, and nucleocapsid proteins, whereas RdRp-focused studies were minimal. Therefore, the primary objective of the meta-analysis was to identify RdRp peptides in experimental data and explore peptide sequence variation among publicly available RdRp protein sequences of SARS-CoV-2, in order to ensure the appropriateness of the SARS-CoV-2 RdRp protein as a drug target. The identified peptides from cell-line and patient proteome data that used RdRp reference sequences were precisely matched with the publicly extracted RdRp sequence. In RdRp sequence analysis, most amino acids were found to be highly conserved across the dataset, except for a few amino acid substitutions that may represent sequencing errors, rather than biological variations. Therefore, a consensus sequence was generated at a 50% sequence cut-off, and one RdRp sequence variation was found at the 323 position, where proline was replaced by leucine (P323L). The effect of the P323L mutation was evaluated on two available RdRp protein structures (7BV1 and 7BV2). An insignificant structural deviation (RMSD: 0.574 Å) was observed when both structures were compared (Supplementary Figure S1B). Our analysis revealed that the P323L mutation decreases the molecular flexibility and makes the mutant protein more stable than the wild type (ΔΔG for the P323L mutant; 7BV1:0.530 kcal/mol and 7BV2:0.460 kcal/mol). The P323L mutation was reported to enhance protein stability in various research studies [40,47]. FP and REM drugs were considered a positive control for exploring the inhibiting potential of TP-derivative compounds. Therefore, an REM-bound RdRp protein structure (7BV2) was used for the subsequent analysis due to the known experimental information regarding the complex. Additionally, the genomic conservation of RdRp was also explored through a phylogenetic analysis of RdRp sequences among six respiratory disease-causing viruses. In this study, the evolutionary conservation of the SARS-CoV-2 RdRp protein was analysed by a protein sequences comparison, and the result revealed that RdRp proteins had a high sequence conservation during evolutionary history.

The present study evaluated the in silico binding efficacy of TP-natural and synthetic derivatives against SARS-CoV-2 RdRp. Additionally, comparative studies of ligand efficacies were performed in two ways: (i) between SARS-CoV-2 RdRp and SARS-CoV RdRp, and (ii) with Remdesivir and Favipiravir drugs. These compounds can inhibit the viral polymerases by mimicking the binding of nucleoside substrates. Regarding 5-methyl-6-nitro-1,2,4-triazolo[1,5-a]pyrimidin-7-one monohydrate, (synthetic TP derivative similar to Comp-1 in chemical strucutre), its L-arginine salt was granted permission (Russian patent: RU2586283C1) to treat severe viral infections in Russia, and it has shown an impressive pharmacological profile for antiviral activity [37]. Although vaccines are the first choice of control against SARS-CoV-2 [48], triazolopyrimidine-derivative compounds can help to treat COVID-19.

In the docking studies of the RdRp protein and four TP-based ligands—Comp-1, TPP-1, EMC-1 and TBP-2—the ligands EMC-1 and Comp-1 showed a strong interaction with Lys798, Glu167 and Ser795 residues (Figure 3, Figures S3 and S4) of the RdRp binding pocket. A recent study [14] claimed that Lys798 residue stabilises the core structure of the RdRp substrate-binding domain (Figure S3); the same was corroborated in the current study. In another study using Cordycepin as an inhibitor against SARS-CoV-2, RdRp was observed to interact with the residues of Lys798 and Asp618 at its binding site, which is comparable to the binding site interactions of EMC-1 [49]. Hence, the binding of the TP-based ligands with RdRp can hinder its structural stability and enzymatic activity. These results support the usage of natural TP derivatives as inhibitors of SARS-CoV-2 RdRp. In addition, these ligands have a profile comparable to the already known drugs, Favipiravir and Remdesivir. Favipiravir has a structural similarity with Comp-1, a spatially similar binding site and good total energies. Although REM is structurally different, its binding affinity is slightly higher than Comp-1 and EMC-1. However, Remdesivir has a high molecular weight of 602.585 mg mol^−1^ and a half-life of ~1 hour, which measn that frequent or high doses of the drug are required. This might affect liver functioning and cause hepatotoxicity. Studies from all over the world reported hepatotoxicity cases for Remdesivir [50]. Hence, the identification of a new compound against SARS-CoV-2 is the demand of the present time [51]. In this regard, triazolopyrimidine derivatives are not yet reported to have any adverse events. The toxicity of the drugs used in the current study was analysed using admetSAR2 [52], and no concerning levels of toxicity were predicted (Supplementary File S3). Therefore, the usage of the TP-based ligand can be increased to further study phases.

Although all four compounds show good binding properties, the ligand of interest will be the one that consistently exhibits the best binding affinity and total energy with the RdRp protein. In this respect, both EMC-1 and Comp-1 can be taken further as lead molecules. Moreover, Comp-1 was also observed to have a high binding potential with other SARS-CoV-2 non-structural proteins (Figure 4, Table S4). Hence, it is possible that Comp-1 can bind and inhibit multiple targets, along with the RdRp protein. This is advantageous because targeting multiple proteins simultaneously would improve virus inhibition and reduce the chances of drug resistance [53]. Therefore, it is suggested that mono-methylated triazolopyrimidine can act as a novel lead molecule against the SARS-CoV-2 RdRp protein for COVID-19 treatment. Further, MD simulation reinforced the molecular docking results with comprehensive RMSD, RMSF and Rg profiles (Figure 5, Figure 6 and Figure 7) of ligand-bound and apo forms of RdRp. After the binding of TP ligands with the SARS-CoV-2 RdRp, molecular deviations and fluctuations in protein complexes were reduced, which shows the stable physical conformation and intermolecular interactions established after the initial binding of the TP analogues.

In our study, a natural derivative of TP, Comp-1, is found as to be the most effective ligand against both SARS-CoV-2 and SARS-CoV RdRp proteins, whereas EMC-1 has a better fit with the SARS-CoV-2 RdRp protein. Hence, Comp-1 and EMC-1, both TP compounds, can be repurposed as antiviral agents against COVID-19 virus.

## 5. Conclusions

The emergence of new viral strains and COVID-19 reinfection cases worldwide has encouraged scientists to discover potential therapeutic agents against SARS-CoV-2 infection. The RdRp/nsp12 of the coronavirus is the most recommended and exploited protein target for inhibiting the viral transcription mechanism. The present study demonstrated the inhibition potential of 1,2,4-triazolopyrimidine and its natural derivatives (Comp-1 and EMC-1) against the viral RdRp protein targets (both SARS-CoV-2 and SARS-CoV RdRp protein), along with other non-structural proteins involved in viral transcription, replication and packaging. The molecular docking and dynamics simulation results designated monomethylated triazolopyrimidine and essramycin as potential inhibitors of SARS-CoV-2 RdRp. The comparative studies with Favipiravir and Remdesivir also support our study’s hypothesis. Nonetheless, for safety concerns and drug development, the proposed lead molecules require further concrete in vitro validation, which will lead to the development of a promising drug molecule to combat the COVID-19 pandemic.

## Figures and Tables

**Table 1:** Identification of SARS-CoV-2 RdRp peptides found in infected human cell lines and patient proteomics samples. A detailed description of peptides is provided in Supplementary Table S2.

Project ID	Cell Line/Patient Sample	Positions	Accession ID	Reference
PXD018581	Lung carcinoma cell line	4452–4490	QPM30612.1	[33]
		4491–4508	QOE87934.1	
		4576–4589	QMS52714.1	
		4926–4945	QOF20355.1	
		4976–4995	QLC46995.1	
		5214–5241	QNO75717.1	
		5214–5250	QPF53892.1	
PXD017710	Colon carcinoma cell line	4525–4552	QKJ68603.1	[34]
		4831–4870	QNO32046.1	
		4929–4947	QOL77454.1	
PXD021328	Naso and oropharyngeal swabs	4405–4410	QQD64054.1	[35]
		4426–4442	QKR84563.1	

**Table 2:** Binding affinity and total energies of ligands docked with SARS-CoV-2 and SARS-CoV RdRps.

Ligand Structure	Ligand Name (Abbreviation)	SARS-CoV-2		SARS-CoV	
		Binding Affinity	Total Energy	Binding Affinity	Total Energy
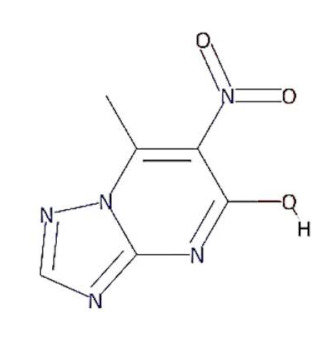	Monomethylated triazolopyrimidine (Comp-1)	−6.134	−8.308	−6.314	−13.864
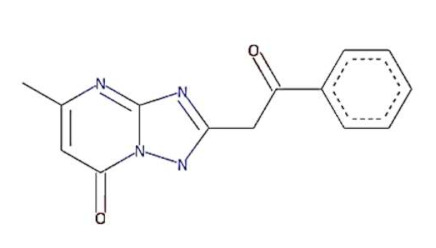	Essramycin (EMC-1)	−6.283	−5.416	−7.039	−6.524
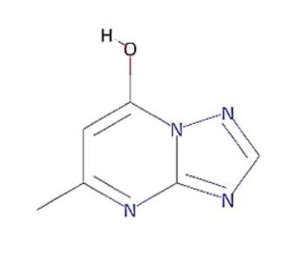	Comp-1 stereoisomer (TBP-2)	−6.15	−8.063	−6.062	−8.291
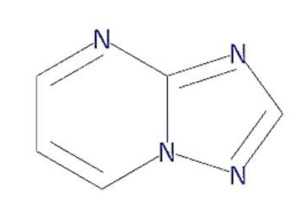	Triazolopyrimidine (TPP-1)	−7.255	−3.689	−6.681	−4.508
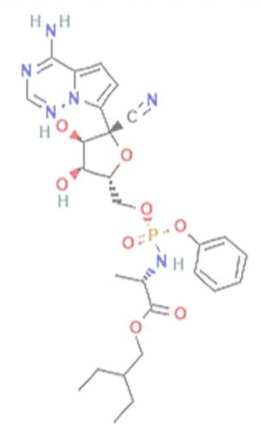	Remdesivir (REM)	−6.995	−30.924	−7.273	−26.782
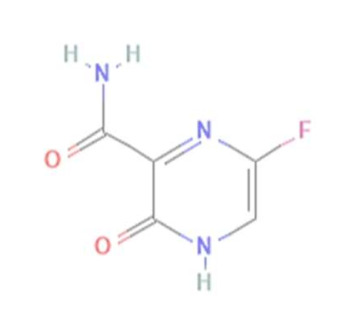	Favipiravir (FP)	−5.793	−6.065	−5.998	−3.678
Units of the energies are kcal/mol					

**Table 3:** Molecular docking results of Comp-1 with SARS-CoV-2 non-structural proteins.

Serial No.	SARS-CoV-2 Protein (PDB ID)	Affinity	Total Energy
1	NSP5/Main Protease (5RHB)	−6.031	−5.596
2	5RLJ/NSP13/Helicase	−6.475	−8.296
3	6WTC/NSP8	−6.689	−5.693
4	7CZ4/NSP3	−6.996	−7.21
5	7K7P/NSP1	−6.508	−4.72
6	7KEH/NSP15	−6.887	−13.277
7	7MSX/NSP2	−6.69	−7.684
All energies are in kcal/mol.			

## Data Availability

Used proteomics data is accessible at ProteomeXchange public repository (PXD017710, PXD018581, and PXD021328). SARS-CoV-2 proteome and RdRp protein sequences were download from NCBI.

## Supplementary Materials

Supplementary File S1: Table S1. Literature survey of transcriptomics studies focused on the expression of SARS-CoV-2 RdRp in infected human cell-line and patient samples. Table S2. Identification of SARS-CoV-2 RdRp peptides found in infected human cell-line and patient samples. Table S3. Viral RdRp amino acid sequences used for phylogenetic analysis. Table S4. Molecular docking results of Comp-1 and EMC-1 with other available SARS-CoV-2 non-structural proteins. Table S5. Molecular docking results of drugs studied against mutated SARS-CoV-2 RdRp (P323L). Figure S1A. Superimposed structures of SARS-CoV-2 and SARS-CoV RdRp. (a) 3D Structure of SARS-CoV, (b) 3D Structure of SARS-CoV-2, and (c) superimposed conformation of SARS-CoV & SARS-CoV-2 RdRp. Figure S1B. Superimposed conformation of 7bv1 and 7bv2 RdRp protein of SARS-CoV-2. (a) Showing the apo RdRp (7bv1), (b) represent the complex RdRp (7bv2) and (c) superimposed structure of both proteins. Figure S2. 3D representation of SARS-CoV-2 RdRp interactions with (A) Favipiravir (B) Remdesivir. Figure S3. 2D representation of ligand interactions with SARS-CoV-2 RdRp. (A) Comp-1 (B) EMC-1 (C) TBP-2 (D) TPP-1. Figure S4. 2D representation of ligand interactions with SARS-CoV RdRp. (A) Comp-1 (B) EMC-1 (C) TBP-2 (D) TPP-1. Figure S5. 2D representation of Comp-1 interactions with SARS-CoV-2 NSPs. (A) NSP1 (B) NSP2 (C) NSP3 (D) NSP5 (E) NSP8 (F) NSP13 (G) NSP15. Figure S6. RMSD values of SARS-CoV-2 RdRp and complexes at (A) set the random velocity of −1 (B) set the random velocity of −2 during 100 ns MD simulation. Figure S7. RMSF values of SARS-CoV-2 RdRp and complexes at (A) set the random velocity of −1 (B) set the random velocity of −2 during 100 ns MD simulation. Figure S8. Rg values of SARS-CoV-2 RdRp and complexes at (A) set the random velocity of −1 (B) set the random velocity of −2 during 100 ns MD simulation. Figure S9. Number of H bonds formed by SARS-CoV-2 RdRp-ligand complexes at (A) set the random velocity of −1 (B) set the random velocity of −2 during 100 ns MD simulation. Figure S10. The solvent accessible surface area of SARS-CoV-2 RdRp and complexes at (A) set the random velocity of −1 (B) set the random velocity of −2 during 100 ns MD simulation. Supplementary File S2: Multiple Sequence Alignment of SARS-CoV-2 RdRP protein. Supplementary File S3: admetSAR2 analysis of drugs used in this study.

## Sample Availability

Samples of the compounds are available from the authors.
