# Supplementary material for: Computational Analysis Reveals Monomethylated Triazolopyrimidine as a Novel Inhibitor of SARS-CoV-2 RNA-Dependent RNA Polymerase (RdRp)
Source: Molecules. 2022 Jan 26;27(3):801. doi: 10.3390/molecules27030801 (PMC8840377; doi:10.3390/molecules27030801)
Supplement: Supplementary file 1 [file molecules-27-00801-s001.zip › Supplementary_FileS1.pdf]

Supplementary Material

Table S1. Literature survey of transcriptomics studies focused on expression of SARS-CoV-2 RdRp in infected human cell line and patient samples.

Cell Line/ Patient Sample	Method	Reference
Normal human bronchial epithelial (NHBE) and human adenocarcinomic alveolar basal epithelial cells (A549)	RNA-Seq	[29]
Throat/nasal swab grown in *Cercopithecus aethiops* kidney epithelial cells (Vero CCl-81)	RT-qPCR	[30]
Throat swab samples	RT-qPCR	[31]
Human lung and colon samples	RT-qPCR	[32]
RT-qPCR = reverse transcriptase quantitative PCR; RNA-Seq = ribonucleic acid sequencing		

Table S2. Identification of SARS-CoV-2 RdRp peptides found in infected human cell line and patient samples.

Project ID	Cell Line/ Patient Sample	Peptide sequences	Positions	Accession ID
PXD017710	Colan carcinoma cell line	HDFFKFRIDVDMVPHISR	4491-4508	QOE87934.1
		NVTPTITQMNLKYAISAKNR	4926-4945	QOF20355.1
		DEDDNLIDSYFVVKRPTFSNYQHEETIYNLLKDCPAVAK	4452-4490	QPM30612.1
		GATVLIGTSKFYGGWHNMLK	4976-4995	QLC46995.1
		QCDDYVYLPYPDPSRILGAGCFVDDIVK	5214-5241	QNO75717.1
		QALLKTVQFCDAIR	4576-4589	QMS52714.1
PXD018581	Lung carcinoma cell line	FLPFQQFGRDIADTTDAVR	4929-4947	QOL77454.1
		HFFFAQDGNAAISDYDYYRYNLPTMCDIRQLLFVVEVADK	4831-4870	QNO32046.1
		HFDEGNCGTLKEILVTYNCCDDDYFNKK	4525-4552	QKJ68603.1
PXD019686	Nasal swab simili	AFDIYNDKVAGFAKFLK	4426-4442	QOQ08810.1
		AFDIYNNKVAGFAKFLK	4426-4442	QKR84563.1

Table S3. Viral RdRp amino acid sequences used for phylogenetic analysis

>SARS-CoV| UniProt ID: P0C6X7 (Severe acute respiratory syndrome coronavirus)<br>SADASTFLNRVCGVSAARLTPCGTGTSTDVVYRAFDIYNEKVAGFAKFLKTNCCRFQEKD<br>EEGNLLDSYFVVKRHTMSNYQHEETIYNLVKDCPAVAVHDFFKFRVDGDMVPHISRQRLT<br>KYTMADLVYALRHFDEGNCDTLKEILVTYNCCDDDYFNKKDWYDFVENPDILRVYANLGE<br>RVRQSLLKTVQFCDAMRDAGIVGVLTLDNQDLNGNWYDFGDFVQVAPGCGVPIVDSYYSL<br>LMPILTLTRALAAESHMDADLAKPLIKWDLLKYDFTEERLCLFDRYFKYWDQTYHPNCIN<br>CLDDRCILHCANFNVLFSTVFPPTSFGPLVRKIFVDGVPFVVSTGYHFRELGVVHNQDVN<br>LHSSRLSFKELLVYAADPAMHAASGNLLLDKRTTCFSVAALTNNVAFQTVKPGNFNKDFY<br>DFAVSKGFFKEGSSVELKHFFFAQDGNAAISDYDYYRYNLPTMCDIRQLLFVVEVVDKYF<br>DCYDGGCINANQVIVNNLDKSAGFPFNKWGKARLYYDSMSYEDQDALFAYTKRNVIPTIT<br>QMNLKYAISAKNRARTVAGVSICSTMTNRQFHQKLLKSIAATRGATVVIGTSKFYGGWHN<br>MLKTVYSDVETPHLMGWDYPKCDRAMPNMLRIMASLVLARKHNTCCNLSHRFYRLANECA<br>QVLSEMVMCGGSLYVKPGGTSSGDATTAYANSVFNICQAVTANVNALLSTDGNKIADKYV<br>RNLQHRLYECLYRNRDVDHEFVDEFYAYLRKHFSMMILSDDAVVCYNSNYAAQGLVASIK<br>NFKAVLYYQNNVFMSEAKCWTETDLTKGPHEFCSQHTMLVKQGDDYVYLPYPDPSRILGA<br>GCFVDDIVKTDGTLMIERFVSLAIDAYPLTKHPNQEYADVFHLYLQYIRKLHDELTGHML<br>DMYSVMLTNDNTSRYWEPEFYEAMYTPHTVLQ
>SARS-CoV-2| UniProt ID: P0DTD1 (Severe acute respiratory syndrome coronavirus 2)<br>SADAQSFLNRVCGVSAARLTPCGTGTSTDVVYRAFDIYNDKVAGFAKFLKTNCCRFQEKD<br>EDDNLIDSYFVVKRHTFSNYQHEETIYNLLKDCPAVAKHDFFKFRIDGDMVPHISRQRLT<br>KYTMADLVYALRHFDEGNCDTLKEILVTYNCCDDDYFNKKDWYDFVENPDILRVYANLGE<br>RVRQALLKTVQFCDAMRNAGIVGVLTLDNQDLNGNWYDFGDFIQTTPGSGVPVVDSYYSL<br>LMPILTLTRALTAESHVDTDLTKPYIKWDLLKYDFTEERLKLFDRYFKYWDQTYHPNCVN<br>CLDDRCILHCANFNVLFSTVFPPTSFGPLVRKIFVDGVPFVVSTGYHFRELGVVHNQDVN<br>LHSSRLSFKELLVYAADPAMHAASGNLLLDKRTTCFSVAALTNNVAFQTVKPGNFNKDFY<br>DFAVSKGFFKEGSSVELKHFFFAQDGNAAISDYDYYRYNLPTMCDIRQLLFVVEVVDKYF<br>DCYDGGCINANQVIVNNLDKSAGFPFNKWGKARLYYDSMSYEDQDALFAYTKRNVIPTIT<br>QMNLKYAISAKNRARTVAGVSICSTMTNRQFHQKLLKSIAATRGATVVIGTSKFYGGWHN<br>MLKTVYSDVENPHLMGWDYPKCDRAMPNMLRIMASLVLARKHTTCCSLSHRFYRLANECA

QVLSEMVMCGGSLYVKPGGTSSGDATTAYANSVFNICQAVTANVNALLSTDGNKIADKYV<br>RNLQHRLYECLYRNRDVDTDFVNEFYAYLRKHFSMMILSDDAVVCFNSTYASQGLVASIK<br>NFKSVLYYQNNVFMSEAKCWTETDLTKGPHEFCSQHTMLVKQGDDYVYLPYPDPSRILGA<br>GCFVDDIVKTDGTLMIERFVSLAIDAYPLTKHPNQEYADVFHLYLQYIRKLHDELTGHML<br>DMYSVMLTNDNTSRYWEPEFYEAMYTPHTVLQ
>MERS| UniProt ID: K9N7C7 (Middle East respiratory syndrome-related coronavirus)<br>SKDSNFLKRVRGSIVNARIEPCSSGLSTDVVFRAFDICNYKAKVAGIGKYYKTNTCRFVE<br>LDDQGHHLDSYFVVKRHTMENYELEKHCYDLLRDCDAVAPHDFFIFDVDKVKTPHIVRQR<br>LTEYTMMDLVYALRHFDQNSEVLKAILVKYGCCDVTYFENKLWFDFVENPSVIGVYHKLG<br>ERVRQAILNTVKFCDHMVKAGLVGVLTLDNQDLNGKWYDFGDFVITQPGSGVAIVDSYYS<br>YLMPVLSMTDCLAAETHRDCDFNKPLIEWPLTEYDFTDYKVQLFEKYFKYWDQTYHANCV<br>NCTDDRCVLHCANFNVLFAMTMPKTCFGPIVRKIFVDGVPFVVSCGYHYKELGLVMNMDV<br>SLHRHRLSLKELMMYAADPAMHIASSNAFLDLRTSCFSVAALTTGLTFQTVRPGNFNQDF<br>YDFVVSKGFFKEGSSVTLKHFFFAQDGNAAITDYNYYSYNLPTMCDIKQMLFCMEVVNKY<br>FEIYDGGCLNASEVVVNNLDKSAGHPFNKFGKARVYYESMSYQEQDELFAMTKRNVIPTM<br>TQMNLKYAISAKNRARTVAGVSILSTMTNRQYHQKMLKSMAATRGATCVIGTTKFYGGWD<br>FMLKTLYKDVDNPHLMGWDYPKCDRAMPNMCRIFASLILARKHGTCCTTRDRFYRLANEC<br>AQVLSEYVLCGGGYYVKPGGTSSGDATTAYANSVFNILQATTANVSALMGANGNKIVDKE<br>VKDMQFDLYVNVYRSTSPDPKFVDKYYAFLNKHFSMMILSDDGVVCYNSDYAAKGYIAGI<br>QNFKETLYYQNNVFMSEAKCWVETDLKKGPHEFCSQHTLYIKDGDDGYFLPYPDPSRILS<br>AGCFVDDIVKTDGTLMVERFVSLAIDAYPLTKHEDIEYQNVFWVYLQYIEKLYKDLTGHM<br>LDSYSVMLCGDNSAKFWEEAFYRDLYSSPTTLQ
>Rhinovirus| UniProt ID: P23008 (Human rhinovirus 1A (HRV-1A))<br>GQIKVNKHATECGLPTIHTPSKTKLQPSVFYDVFPGSKEPAVLTDNDPRLEVNFKEALFS<br>KYKGNVECNLNEHMEIAIAHYSAQLMTLDIDSRPIALEDSVFGIEGLEALDLNTSAGFPY<br>VTMGIKKRDLINNKTKDISRLKEALDKYGVDLPMITFLKDELRKKEKISTGKTRVIEASS<br>INDTILFRTTFGNLFSKFHLNPGVVTGSAVGCDPETFWSKIPVMLDGDCIMAFDYTNYDG<br>SIHPVWFQALKKVLENLSFQSNLIDRLCYSKHLFKSTYYEVAGGVPSGCSGTSIFNTMIN<br>NIIIRTLVLDAYKNIDLDKLKIIAYGDDVIFSYKYTLDMEAIANEGKKYGLTITPADKSN<br>EFKKLDYSNVTFLKRGFKQDERHTFLIHPTFPVEEIHESIRWTKKPSQMQEHVLSLCHLM<br>WHNGRKVYEDFSSKIRSVSAGRALYIPPYDLLKHEWYEKF
>RSV_Strain_B| UniProt ID: O36635 (Human respiratory syncytial virus B (strain B1))

MDPIINGNSANVYLTDSYLKGVISFSECNALGSYLFNGPYLKNDYTNLISRQSPLLEHMN
LKKLTITQSLISRYHKGELKLEEPTYFQSLLMTYKSMSSSEQIATTNLLKKIIRRAIEIS
DVKVYAILNKLGLKEKDRVKPNNNSGDENSVLTTIIKDDILSAVESNQSYTNSDKNHSVN
QNITIKTTLLKKLMCSMQHPPSWLIHWFNLYTKLNNILTQYRSNEVKSHGFILIDNQTLS
GFQFILNQYGCIVYHKGLKKITTTTYNQFLTWKDISLSRLNVCLITWISNCLNTLNKSLG
LRCGFNNVVLSQLFLYGDCILKLFHNEGFYIIKEVEGFIMSLILNITEEDQFRKRFYNSM
LNNITDAAIKAQKNLLSRVCHTLLDKTVSDNIINGKWIILLSKFLKLIKLAGDNNLNNLS
ELYFLFRIFGHPMVDERQAMDAVRINCNETKFYLLSSLSTLRGAFIYRIIKGFVNTYNRW
PTLRNAIVLPLRWLNYYKLNTYPSLLEITENDLIILSGLRFYREFHLPKKVDLEMIINDK
AISPPKDLIWTSFPRNYMPSHIQNYIEHEKLKFSESDRSRRVLEYYLRDNKFNECDLYNC
VVNQSYLNNSNHVVSLTGKERELSVGRMFAMQPGMFRQIQILAEKMIAENILQFFPESLT
RYGDLELQKILELKAGISNKSNRYNDNYNNYISKCSIITDLSKFNQAFRYETSCICSDVL
DELHGVQSLFSWLHLTIPLVTIICTYRHAPPFIKDHVVNLNEVDEQSGLYRYHMGGIEGW
CQKLWTIEAISLLDLISLKGKFSITALINGDNQSIDISKPVRLIEGQTHAQADYLLALNS
LKLLYKEYAGIGHKLKGTETYISRDMQFMSKTIQHNGVYYPASIKKVLRVGPWINTILDD
FKVSLESIGSLTQELEYRGESLLCSLIFRNIWLYNQIALQLRNHALCNNKLYLDILKVLK
HLKTFFNLDSIDMALSLYMNLPMLFGGGDPNLLYRSFYRRTPDFLTEAIVHSVFVLSYYT
GHDLQDKLQDLPDDRLNKFLTCVITFDKNPNAEFVTLMRDPQALGSERQAKITSEINRLA
VTEVLSIAPNKIFSKSAQHYTTTEIDLNDIMQNIEPTYPHGLRVVYESLPFYKAEKIVNL
ISGTKSITNILEKTSAIDTTDINRATDMMRKNITLLIRILPLDCNKDKRELLSLENLSIT
ELSKYVRERSWSLSNIVGVTSPSIMFTMDIKYTTSTIASGIIIEKYNVNSLTRGERGPTK
PWVGSSTQEKKTMPVYNRQVLTKKQRDQIDLLAKLDWVYASIDNKDEFMEELSTGTLGLS
YEKAKKLFPQYLSVNYLHRLTVSSRPCEFPASIPAYRTTNYHFDTSPINHVLTEKYGDED
IDIVFQNCISFGLSLMSVVEQFTNICPNRIILIPKLNEIHLMKPPIFTGDVDIIKLKQVI
QKQHMFLPDKISLTQYVELFLSNKALKSGSNINSNLILVHKMSDYFHNAYILSTNLAGHW
ILIIQLMKDSKGIFEKDWGEGYITDHMFINLNVFFNAYKTYLLCFHKGYGKAKLECDMNT
SDLLCVLELIDSSYWKSMSKVFLEQKVIKYIVNQDTSLHRIKGCHSFKLWFLKRLNNAKF
TVCPWVVNIDYHPTHMKAILSYIDLVRMGLINVDKLTIKNKNKFNDEFYTSNLFYISYNF
SDNTHLLTKQIRIANSELEDNYNKLYHPTPETLENISLIPVKSNNSNKPKFCISGNTESI
MMSTFSNKMHIKSSTVTTRFNYSKQDLYNLFPNVVIDRIIDHSGNTAKSNQLYITTSHQT
SLVRNSASLYCMLPWHHVNRFNFVFSSTGCKISIEYILKDLKIKDPSCIAFIGEGAGNLL
LRTVVELHPDIRYIYRSLKDCNDHSLPIEFLRLYNGHINIDYGENLTIPATDATNNIHWS

YLHIKFAEPISIFVCDAELPVTANWSKIIIEWSKHVRKCKYCSSVNRCILIAKYHAQDDI<br>DFKLDNITILKTYVCLGSKLKGSEVYLVLTIGPANILPVFDVVQNAKLIFSRTKNFIMPK<br>KTDKESIDANIKSLIPFLCYPITKKGIKTSLSKLKSVVNGDILSYSIAGRNEVFSNKLIN<br>HKHMNILKWLDHVLNFRSAELNYNHLYMIESTYPYLSELLNSLTTNELKKLIKITGSVLY<br>NLPNEQ
>RSV_Strain_A| UniProt ID: Q9IWW8 (Human respiratory syncytial virus A (strain Long))<br>MDPIINGNSANVYLTDSYLKGVISFSECNALGSYIFNGPYLKNDYTNLISRQNPLIEHMN<br>LKKLNITQSLISKYHKGEIKLEEPTYFQSLLMTYKSMTSLEQIATTNLLKKIIRRAIEIS<br>DVKVYAILNKLGLKEKDKIKSNNGQDEDNSVITTIIKDDILSAVKDNQSHLKADKNHSTK<br>QKDTIKTTLLKKLMCSMQHPPSWLIHWFNLYTKLNNILTQYRSNEVKNHGFILIDNQTLS<br>GFQFILNQYGCIVYHKELKRITVTTYNQFLTWKDISLSRLNVCLITWISNCLNTLNKSLG<br>LRCGFNNVILTQLFLYGDCILKLFHNEGFYIIKEVEGFIMSLILNITEEDQFRKRFYNSM<br>LNNITDAANKAQKNLLSRVCHTLLDKTVSDNIINGRWIILLSKFLKLIKLAGDNNLNNLS<br>ELYFLFRIFGHPMVDERQAMDAVKVNCNETKFYLLSSLSMLRGAFIYRIIKGFVNNYNRW<br>PTLRNAIVLPLRWLTYYKLNTYPSLLELTERDLIVLSGLRFYREFRLPKKVDLEMIINDK<br>AISPPKNLIWTSFPRNYMPSHIQNYIEHEKLKFSESDKSRRVLEYYLRDNKFNECDLYNC<br>VVNQSYLNNPNHVVSLTGKERELSVGRMFAMQPGMFRQVQILAEKMIAENILQFFPESLT<br>RYGDLELQKILELKAGISNKSNRYNDNYNNYISKCSIITDLSKFNQAFRYETSCICSDVL<br>DELHGVQSLFSWLHLTIPHVTIICTYRHAPPYIRDHNVDLNNVDEQSGLYRYHMGGIEGW<br>CQKLWTIEAISLLDLISLKGKFSITALINGDNQSIDISKPVRLMEGQTHAQADYLLALNS<br>LKLLYKEYAGIGHKLKGTETYISRDMQFMSKTIQHNGVYYPASIKKVLRVGPWINTILDD<br>FKVSLESIGSLTQELEYRGESLLCSLIFRNVWLYNQIALQLKNHALCNNKLYLDILKVLK<br>HLKTFFNLDNIDTALTLYMNLPMLFGGGDPNLLYRSFYRRTPDFLTEAIVHSVFILSYYT<br>NHDLKDKLQDLSDDRLNKFLTCIITFDKNPNAEFVTLMRDPQALGSERQAKITSEINRLA<br>VTEVLSTAPNKIFSKSAQHYTTTEIDLNDIMQNIEPTYPHGLRVVYESLPFYKAEKIVNL<br>ISGTKSITNILEKTSAIDLTDIDRATEMMRKNITLLIRILPLDCNRDKREILSMENLSIT<br>ELSKYVRERSWSLSNIVGVTSPSIMYTMDIKYTTSTIASGIIIEKYNVNSLTRGERGPTK<br>PWVGSSTQEKKTMPVYNRQVLTKKQRDQIDLLAKLDWVYASIDNKDEFMEELSIGTLGLT<br>YEKAKKLFPQYLSVNYLHRLTVSSRPCEFPASIPAYRTTNYHFDTSPINRILTEKYGDED<br>IDIVFQNCISFGLSLMSVVEQFTNVCPNRIILIPKLNEIHLMKPPIFTGDVDIHKLKQVI<br>QKQHMFLPDKISLTQYVELFLSNKTLKSGSHVNSNLILAHKISDYFHNTYILSTNLAGHW<br>ILIIQLMKDSKGIFEKDWGEGYITDHMFINLKVFFNAYKTYLLCFHKGYGKAKLECDMNT

SDLLCVLELIDSSYWKSMSKVFLEQKVIKYILSQDASLHRVKGCHSFKLWFLKRLNVAEF
TVCPWVVNIDYHPTHMKAILTYIDLVRMRLINIDRIHIKNKHKFNDEFYTSNLFYINYNF
SDNTHLLTKHIRIANSELENNYNKLYHPTPETLENILANPIKSNDKKTLNDYCIGKNVDS
IMLPLLSNKKLVKSSAMIRTNYSKQDLYNLFPTVVIDRIIDHSGNTAKYNQLYTTTSHQI
SLVHNSTSLYCMLPWHHINRFNFVFSSTGCKISIEYILKDLKIKDPNCIAFIGEGAGNLL
LRTVVELHPDIRYIYRSLKDCNDHSLPIEFLRLYNGHINIDYGENLTIPATDATNNIHWS
YLHIKFAEPISLFVCDAELPVTVNWSKIIIEWSKHVRKCKYCSSVNKCTLIVKYHAQDDI
DFKLDNITILKTYVCLGSKLKGSEVYLVLTIGPANIFPVFNVVQNAKLILSRTKNFIMPK
KADKESIDANIKSLIPFLCYPITKKGINTALSKLKSVVSGDILSYSIAGRNEVFSNKLIN
HKHMNILKWFNHVLNFRSTELNYNHLYMVESTYPYLSELLNSLTTNELKKLIKITGSLLY
NFHNE

Table S4. Molecular docking results of Comp-1 and EMC-1 with other available SARS-CoV-2 non-structural proteins.

**Protein/Ligand**	**EMC-1**		**Comp-1**		**TBP-2**		**TPP-1**	
**PDB ID_Protein Name**	**Affinity**	**Total Energy**	**Affinity**	**Total Energy**	**Affinity**	**Total Energy**	**Affinity**	**Total Energy**
**5rhb_MPro**	-6.739	-1.884	-6.031	-5.596	-5.923	-6.512	-6.674	-0.538
**5rlj_Helicase**	-7.545	-4.596	-6.475	-8.296	-6.112	-9.81	-6.963	-6.035
**6wtc_NSP8**	-6.474	1.683	-6.689	-5.693	-6.249	-6.705	-6.462	0.056
**7cz4_NSP3**	-8.359	-5.425	-6.996	-7.21	-6.208	-6.488	-7.043	-3.45
**7k7p_NSP1**	-6.769	-0.568	-6.508	-4.72	-6.285	-5.569	-6.57	-2.187
**7keh_NSP15**	-7.332	-2.183	-6.887	-13.277	-6.444	-9.075	-6.667	-3.873
**7msx_NSP2**	-7.607	-1.233	-6.69	-7.684	-6.274	-6.048	-7.295	-6.405

All energies are in kcal/mol. Colour fill indicates the best energies of docked complex.

Table S5. Molecular docking results of drugs studied against mutated SARS-CoV-2 RdRp (P323L).

Ligand	Affinity	Total Energy
**REM**	-7.589	-31.368
**Comp-1**	-6.195	-7.928
**EMC-1**	-6.074	-5.247
**FAV**	-5.901	-4.163
**TPP**	-6.456	-3.135

All energies are in kcal/mol. Colour fill indicates the best energies of docked complex.

EMC-1 = Essramycin
Comp-1 = Monomethylated triazolopyrimidine
TBP-2 = MTP stereoisomer
TPP-1 = Triazolopyrimidine
REM = Remdesivir
FAV = Favipiravir

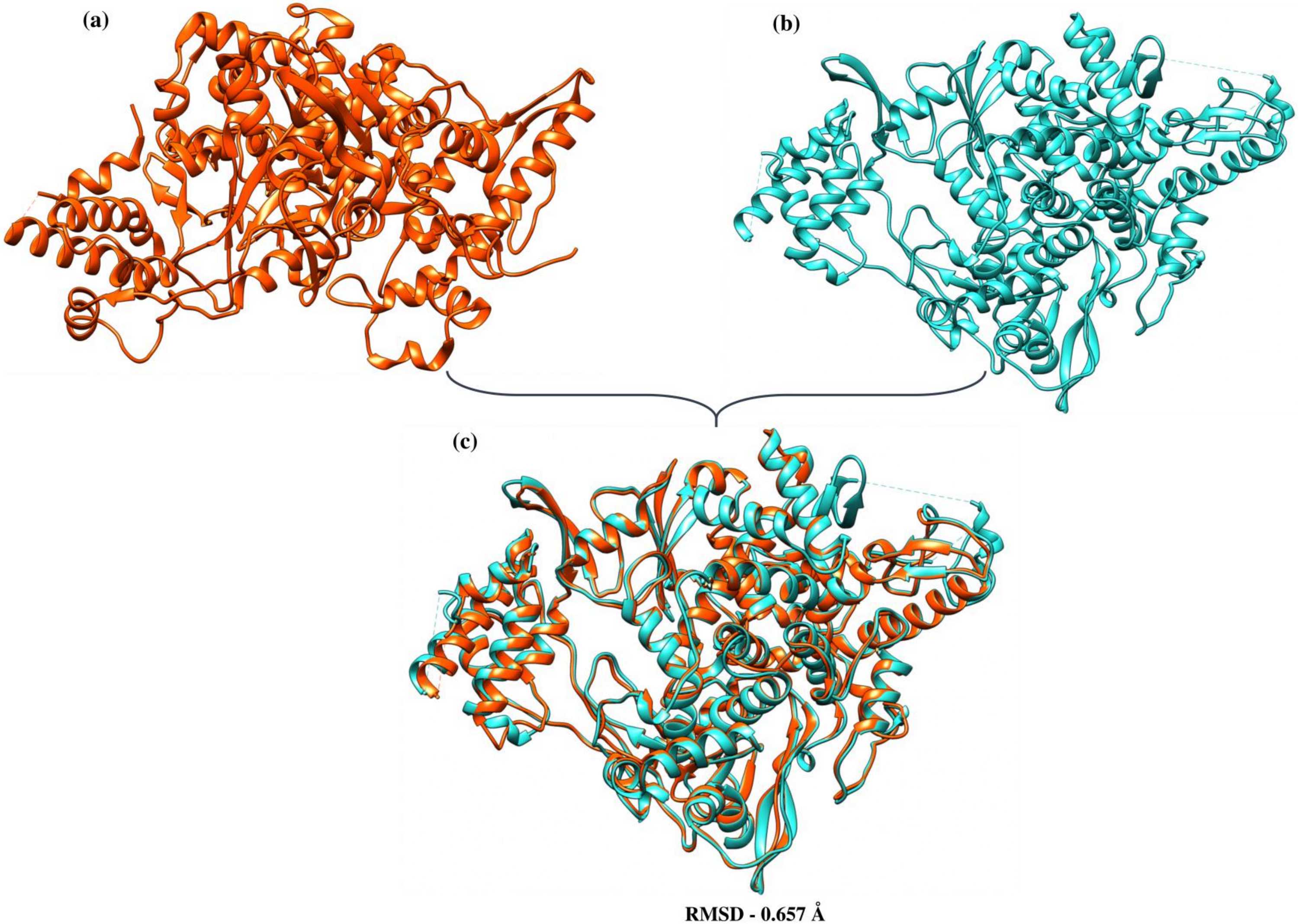

Figure S1(A). Superimposed structures of SARS-CoV-2 and SARS-CoV RdRp. **(a)** 3D structure of SARS-CoV, **(b)** 3D structure of SARS-CoV-2 and **(c)** superimposed conformation of SARS-CoV & SARS-CoV-2 RdRp.

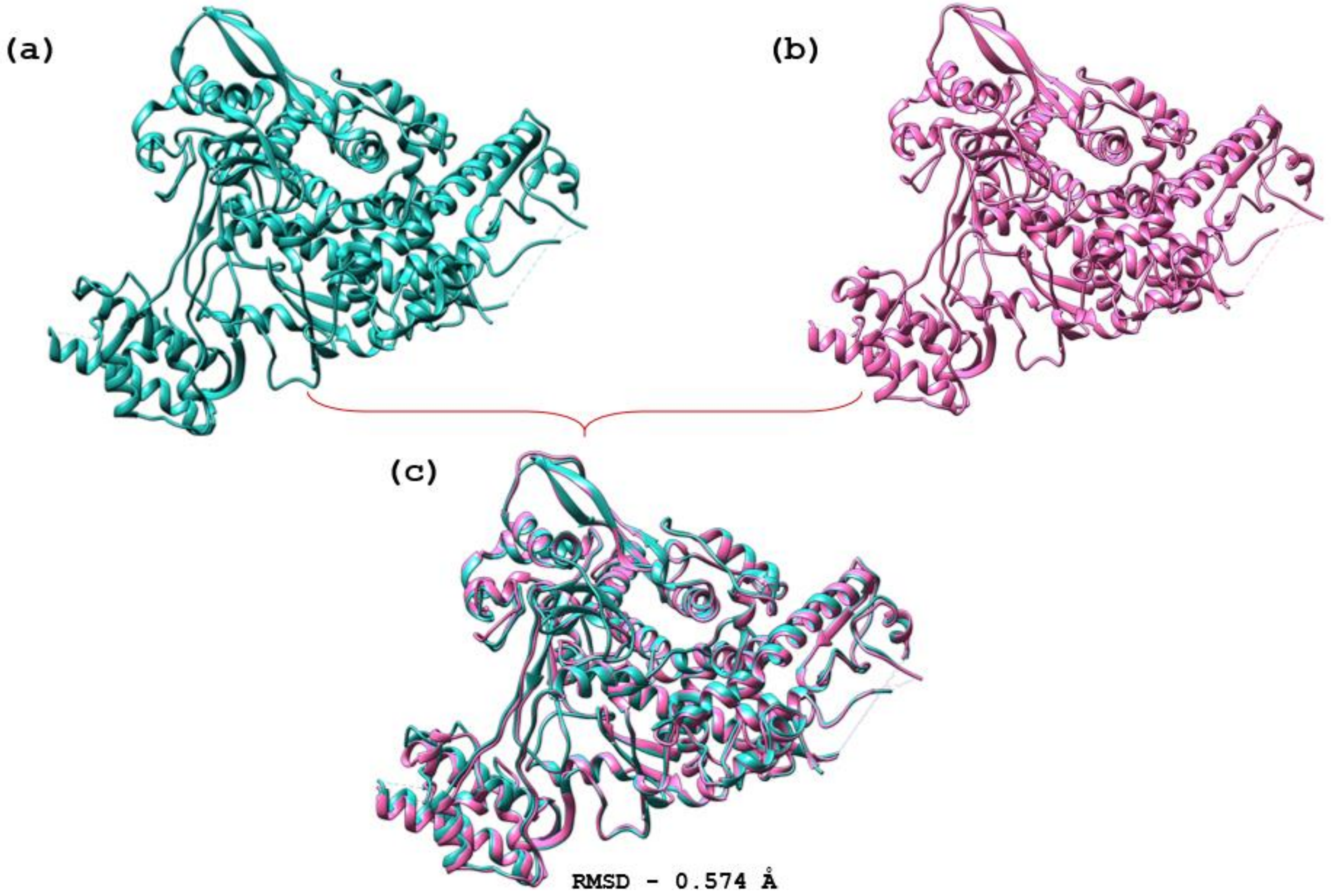

Figure S1 (B). Superimposed conformation of 7bv1 and 7bv2 RdRp protein of SARS-CoV-2. (a) Showing the apo Rdrp (7bv1), (b) represent the complex RdRp (7bv2) and (c) superimposed structure of both protein

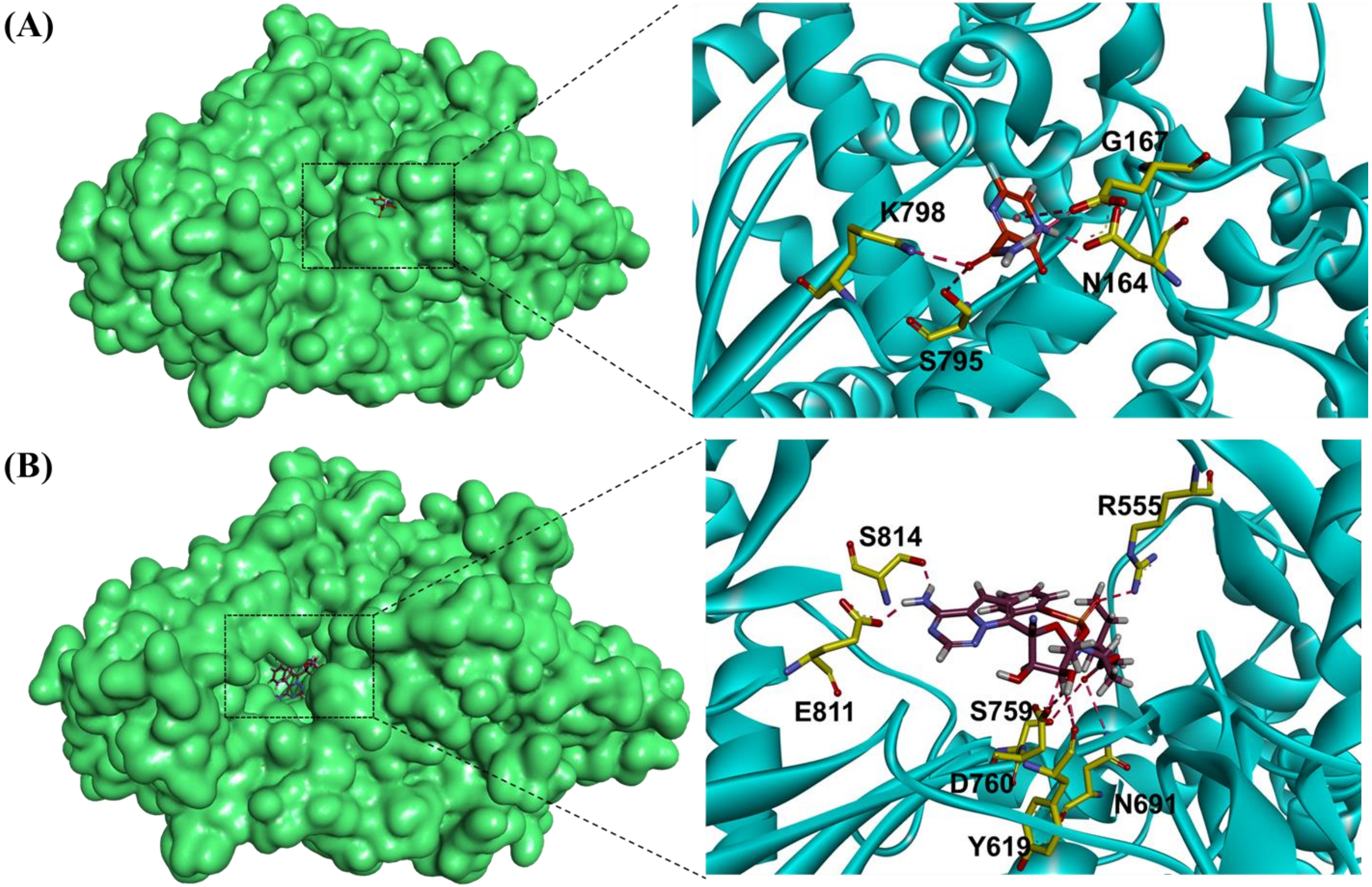

Figure S2. 3D representation of SARS-CoV-2 RdRp interactions with (A) Favipiravir (B) Remdesivir.

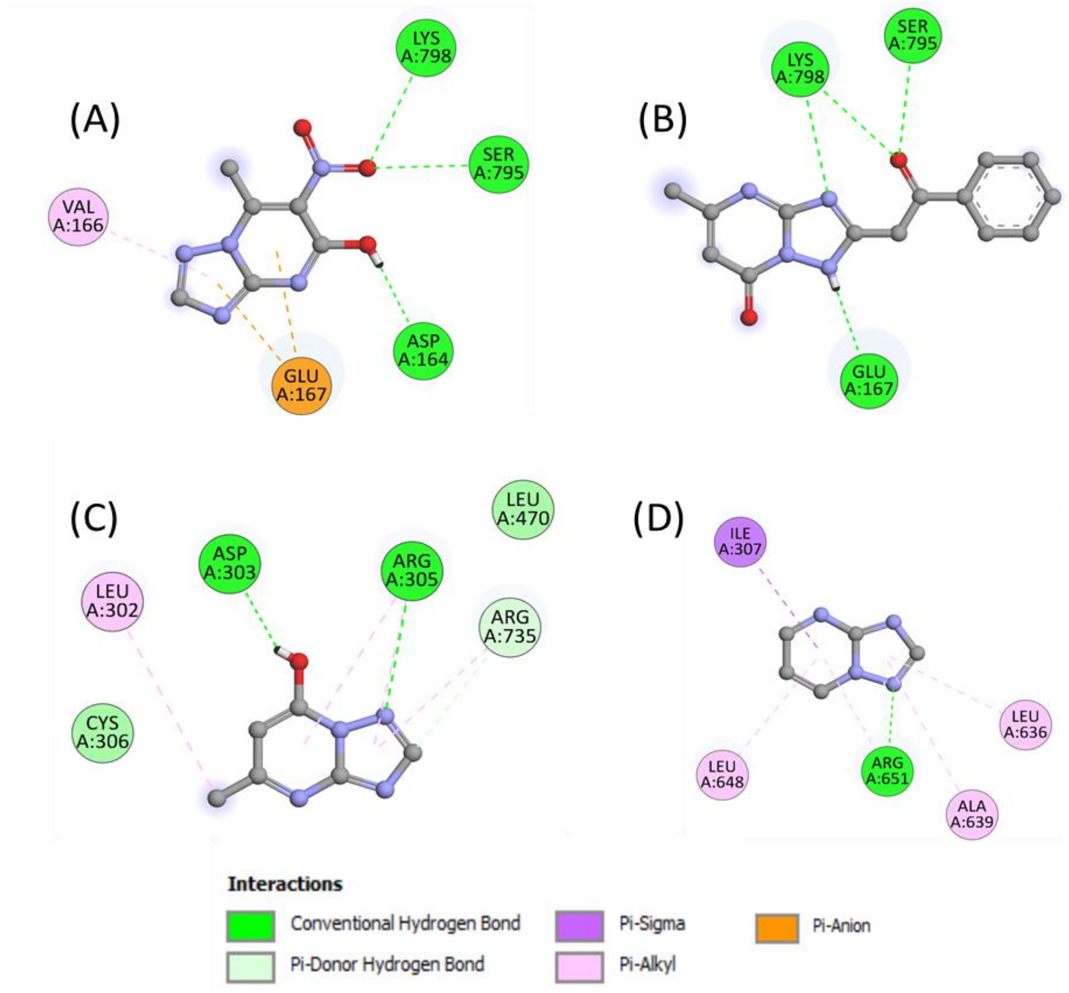

Figure S3. 2D representation of ligand interactions with SARS-CoV-2 RdRp. (A) Comp-1 (B) EMC-1 (C) TBP-2 (D) TPP-1.

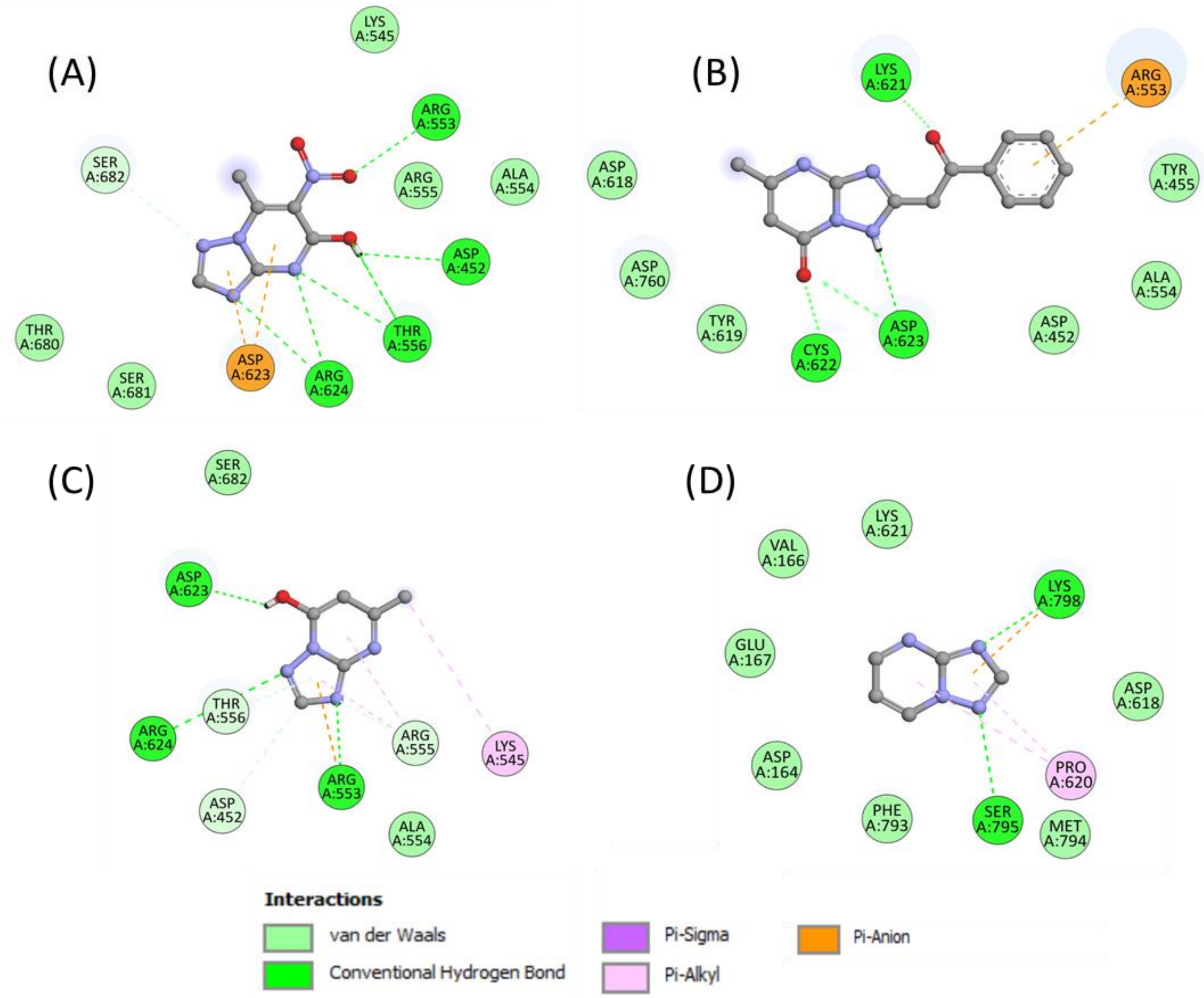

Figure S4. 2D representation of ligand interactions with SARS-CoV RdRp. (A) Comp-1 (B) EMC-1 (C) TBP-2 (D) TPP-1.

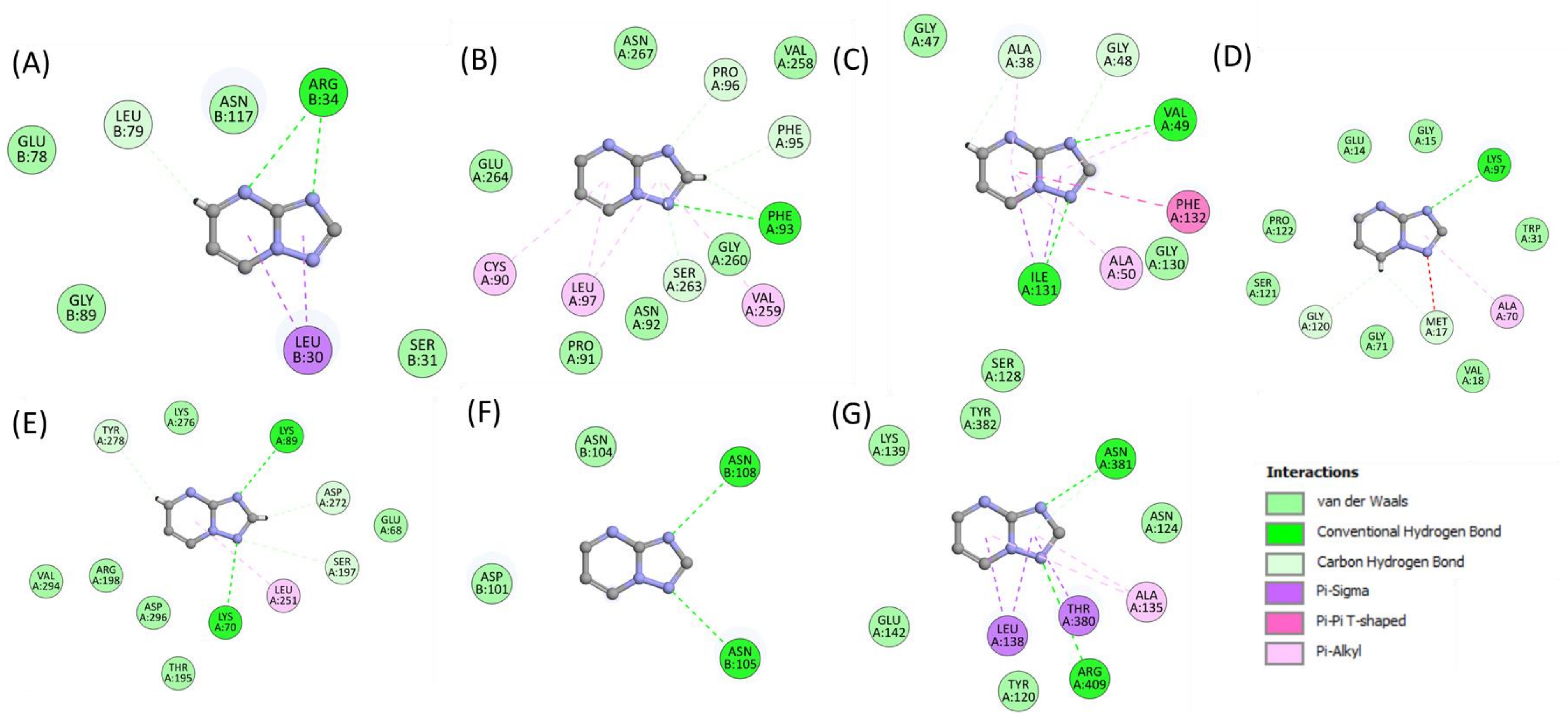

Figure S5. 2D representation of Comp-1 interactions with SARS-CoV-2 NSPs. (A) NSP1 (B) NSP2 (C) NSP3 (D) NSP5 (E) NSP8 (F) NSP13 (G) NSP15.

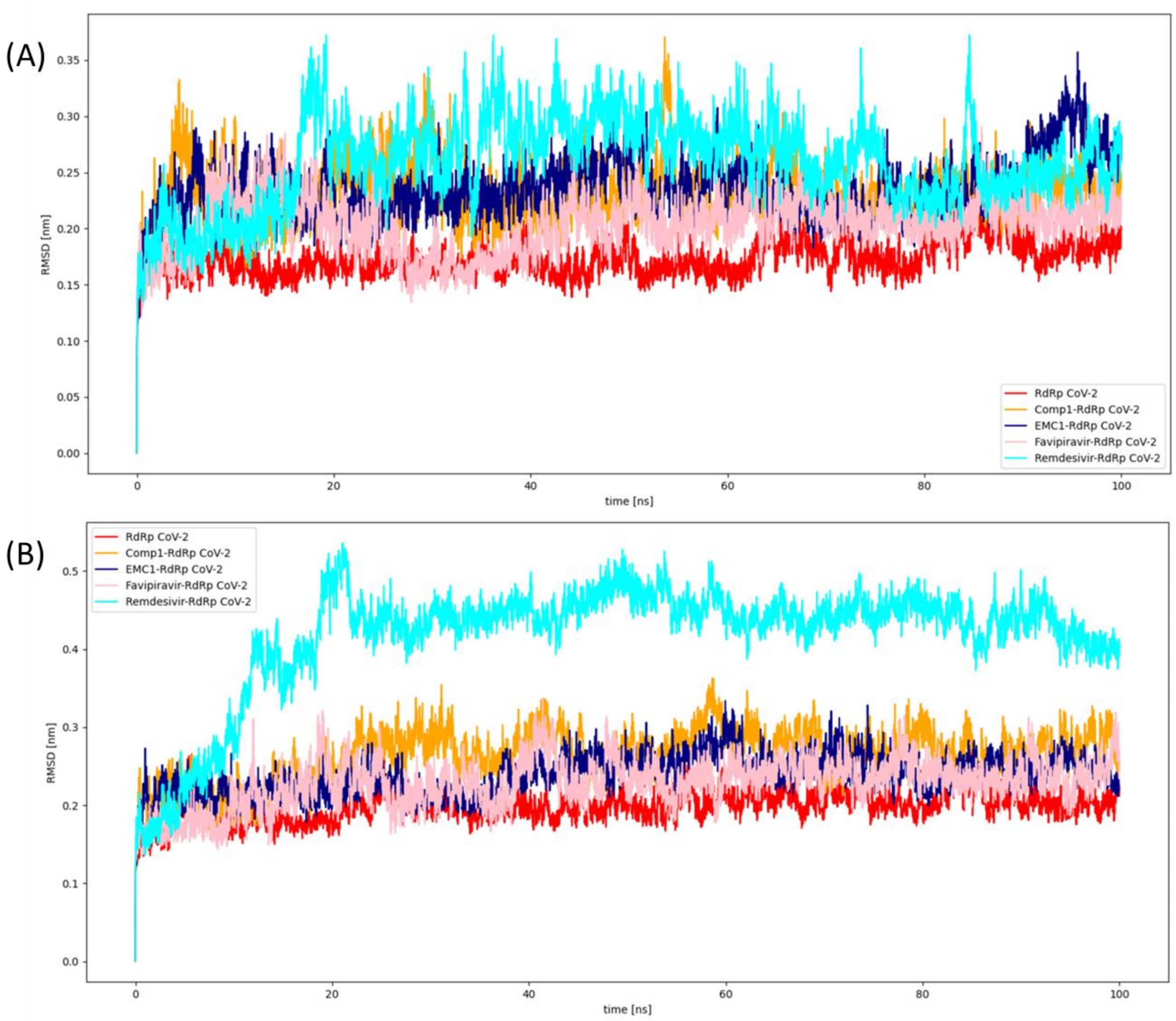

Figure S6. RMSD values of SARS-CoV-2 RdRp and complexes at (A) set random velocity of -1 (B) set random velocity of -2 during 100 ns MD simulation.

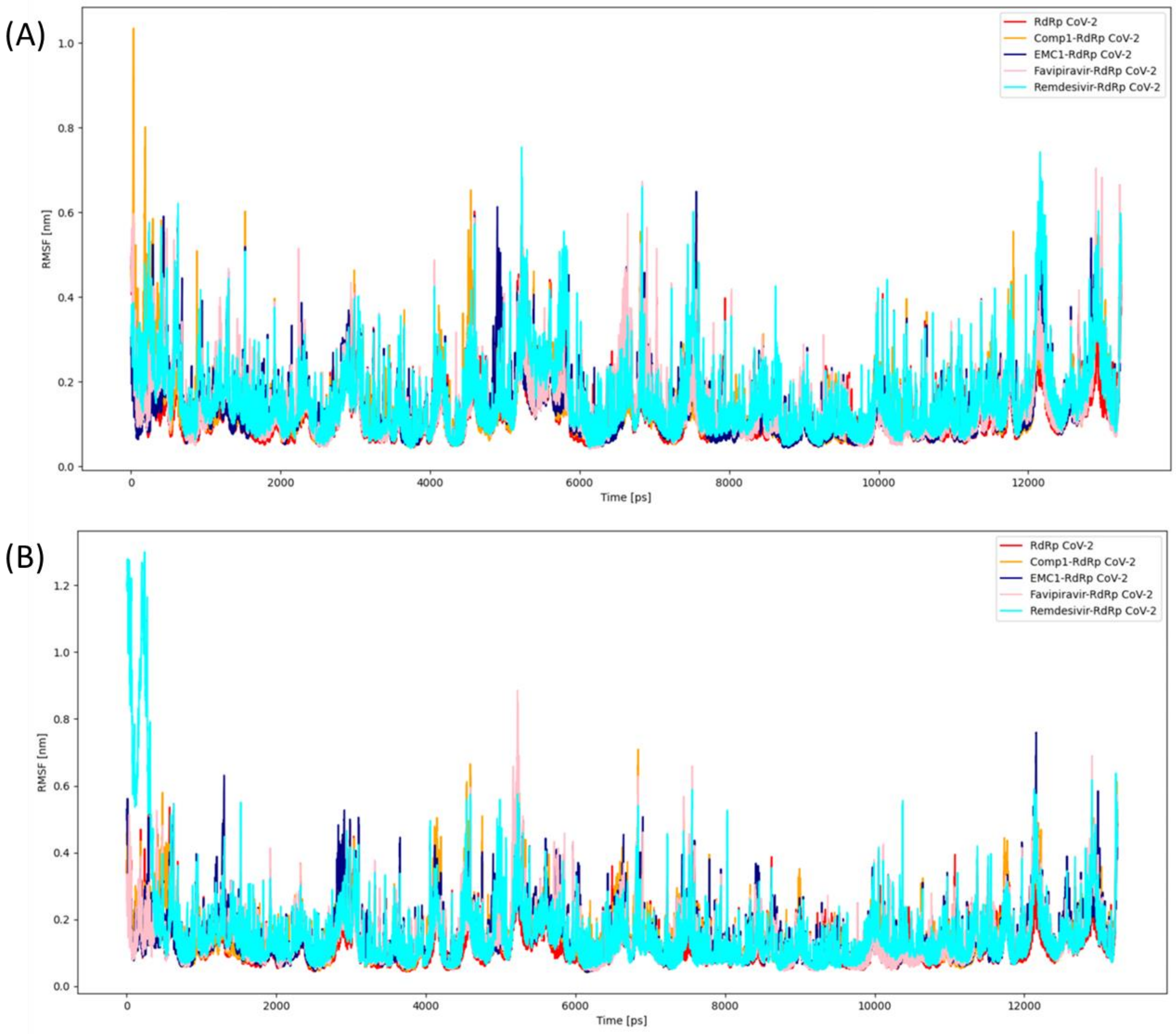

Figure S7. RMSF values of SARS-CoV-2 RdRp and complexes at (A) set random velocity of -1 (B) set random velocity of -2 during 100 ns MD simulation.

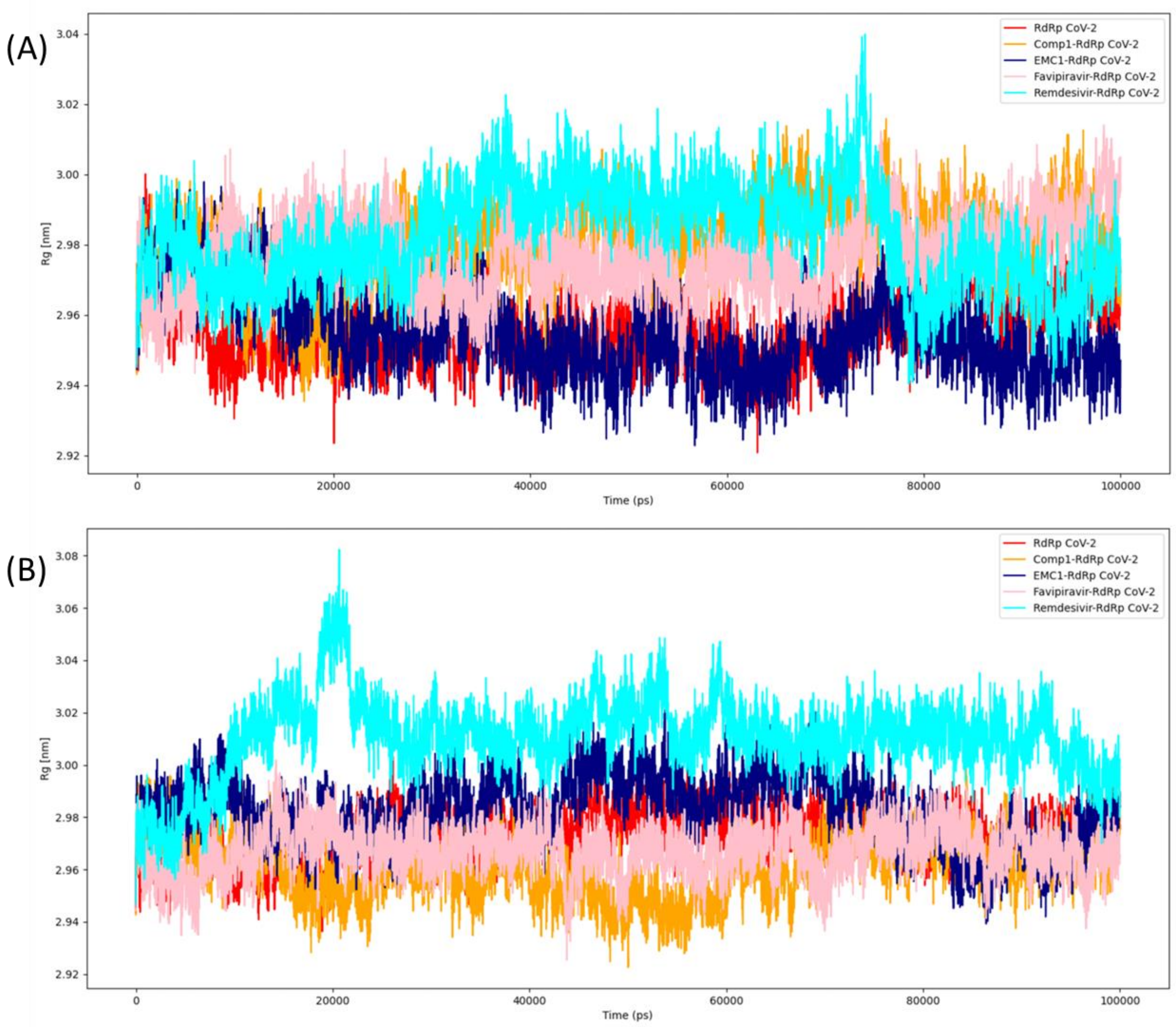

Figure S8. R_g values of SARS-CoV-2 RdRp and complexes at (A) set random velocity of -1 (B) set random velocity of -2 during 100 ns MD simulation.

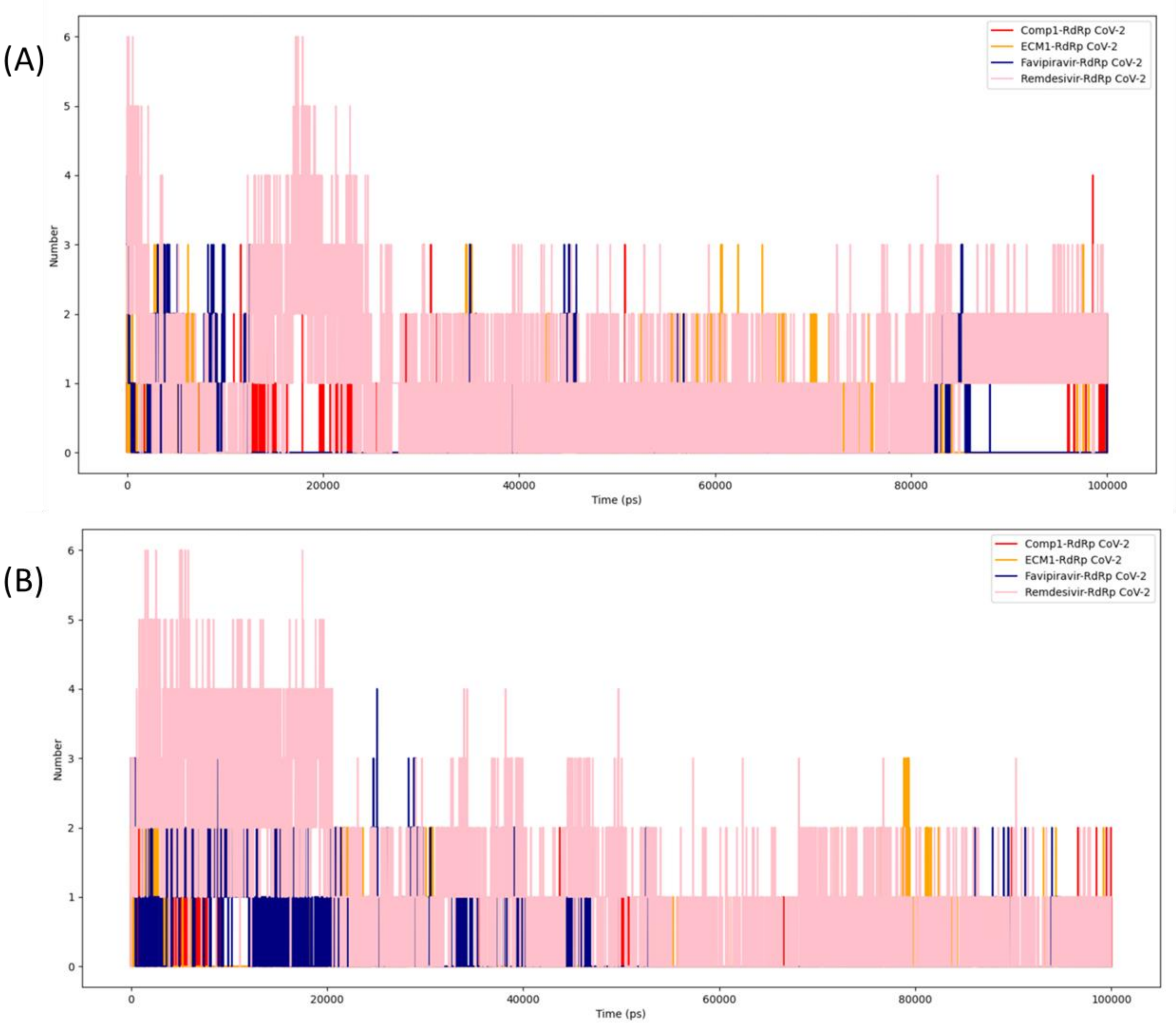

Figure S9. Number of H-bonds formed by SARS-CoV-2 RdRp-ligand complexes at (A) set random velocity of -1 (B) set random velocity of -2 during 100 ns MD simulation.

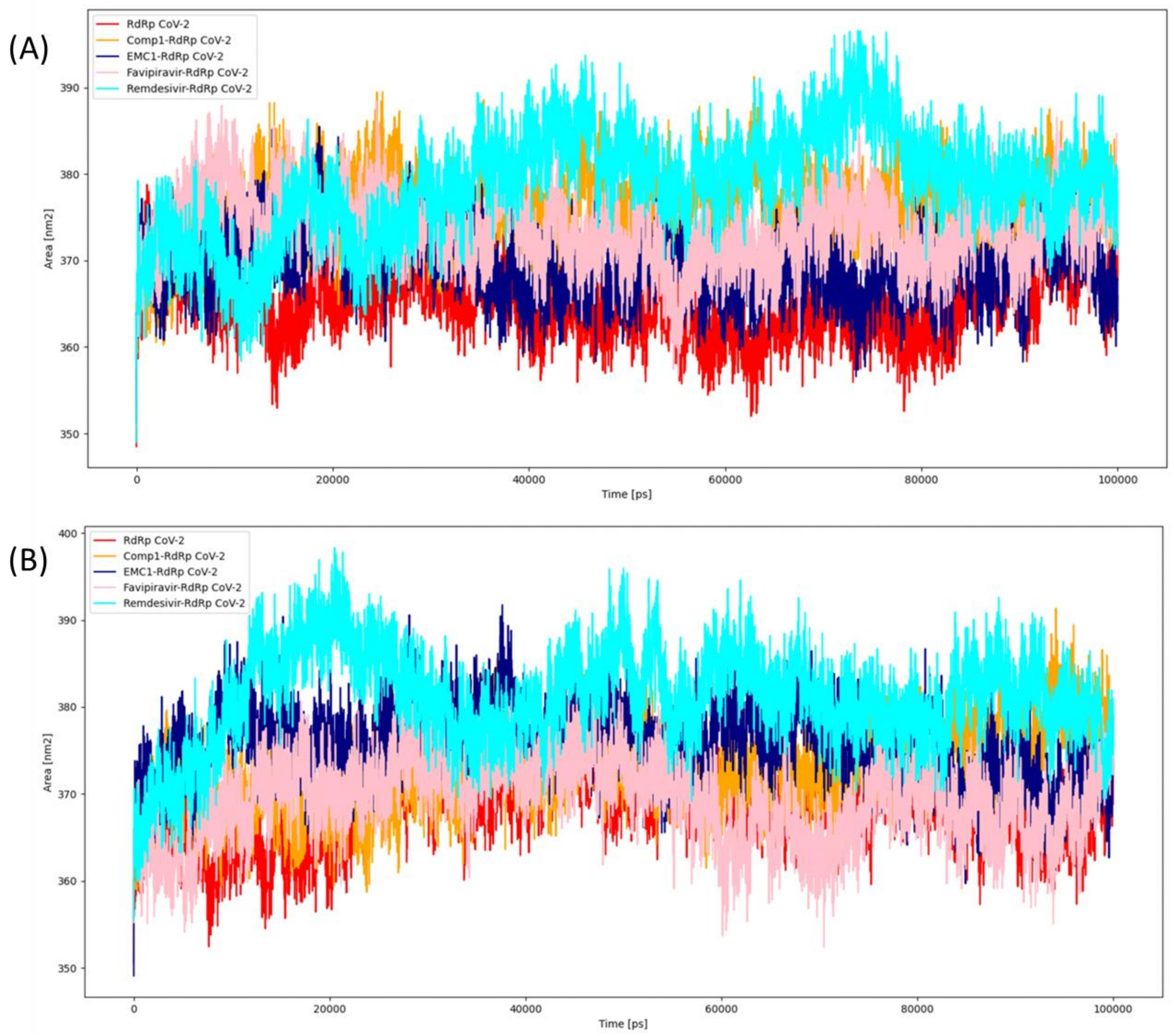

Figure S10. Solvent accessible surface area of SARS-CoV-2 RdRp and complexes at (A) set random velocity of -1 (B) set random velocity of -2 during 100 ns MD simulation.
